# The AF-2 domain of estrogen-related receptor interacts with PPRC1 to modulate larval epidermal marking in Bombyx mori

**DOI:** 10.1371/journal.pgen.1012339

**Published:** 2026-10-12

**Authors:** Jinxin Wu, Die Liu, Haoran Xu, Lingqing Zeng, Ping Lin, Ping Zhao, Guanwang Shen

**Affiliations:** Integrative Science Center of Germplasm Creation in Western China (Chongqing) Science City, Biological Science Research Center, Southwest University, Chongqing, China; University of Kentucky, UNITED STATES OF AMERICA

## Abstract

Estrogen-related receptors (ERRs) have no natural ligands, vertebrate ERRs rely on their activation function-2 (AF-2) structural domain to recruit coactivators for transcriptional regulation. While metabolic regulation in insects differs significantly from that in vertebrates, it remains unclear whether the regulatory function of insect ERR requires coactivators. Using silkworms as the research subject, this study aims to analyze the molecular mechanisms of ERR-regulated metabolism in insects and refine the metabolic regulatory network. This study applied CRISPR/Cas9 gene editing technology to knock out the AF-2 domain of BmERR in the lepidopteran silkworms and examined its effects on functional role, associated interacting factors, and related physiological processes. Disruption of the BmERR AF-2 domain inhibited the expression of melanin metabolism-related genes *BmTH*, *BmDDC*, *BmAA-NAT*, *Bmebony*, and *Bmtan*, leading to the accumulation of tyrosine and dopa, thereby causing aberrant melanin metabolism and loss of epidermal markings. By contrast, BmERR overexpression enhanced the pathway, resulting in intensification of epidermal markings. The homologous gene of the ERR coactivator, BmPPRC1, showed highly consistent expression with BmERR in the epidermis. The immunofluorescence signal of BmPPRC1 was localized to the nucleus and highly overlapped with that of BmERR. BmPPRC1 interacts with the AF-2 domain of BmERR, and deletion of this region weakens the regulation of melanin metabolism-related genes in silkworm embryonic cells. The ERR function and mode of action are evolutionarily conserved, and its AF-2 structural domain binds equally well to the coactivator BmPPRC1, providing new perspectives for studying the modulability of insect mottle formation.

## Author summary

It is known that estrogen-related receptors (ERRs) in vertebrates lack natural ligands and rely on their activation function-2 (AF-2) domain to recruit coactivators for transcriptional regulation. However, insects differ significantly from vertebrates in terms of metabolic regulation, and it remains unclear whether insect ERRs function via coactivators. Therefore, we knocked out the AF-2 domain of ERR in the silkworm and investigated the molecular mechanisms by which ERR regulates insect metabolism. Our results indicate that inhibiting genes related to melanin metabolism leads to the accumulation of tyrosine and DOPA, disrupts melanin production, and results in the loss of epidermal markings. In contrast, overexpression of BmERR enhances this pathway, deepening epidermal markings. We also found that the coactivator homolog BmPPRC1 is co-expressed with BmERR in the epidermis and colocalizes with BmERR in the nucleus. Our finding not only provides fresh insights into the mechanisms of epidermal markings but also broadens the research scope for understanding the biological functions and regulatory networks of ERR and other orphan nuclear receptors in insects.

## 1. Introduction

Nuclear receptors form the largest family of transcriptional regulators in metazoans. They bind to specific DNA response elements in the promoters of downstream target genes as monomers or dimers, thereby controlling gene transcription. These receptors play key roles in a wide range of biological processes, including energy metabolism, cell growth and differentiation, apoptosis, autophagy, development, and the maintenance of organismal homeostasis [1,2]. The estrogen-related receptor (ERR) was the first orphan nuclear receptor discovered. It has a typical nuclear receptor functional structure, including a transcriptional activation function AF-1 region at the N-terminus, a DNA-binding domain, hinge region, and ligand-binding domain at the C-terminus [3,4], which also contains ligand-dependent activation function-2 (AF-2) [5]. Due to the lack of natural ligands, ERR directly binds to the coactivators of other nuclear receptors or transcription factors, thereby regulating the transcription of target genes and participating in energy metabolism and biogenesis in vivo [6,7].

ERR exists in three isoforms in vertebrates (ERRα, ERRβ, and ERRγ) and is an important regulator of vertebrate carcinogenesis, energy metabolism, and mitochondrial biogenesis. It can control the expression of genes related to lipid transport and uptake, tricarboxylic acid cycle, mitochondrial biogenesis, and oxidative phosphorylation [8,9]. In the process of biological evolution, the regulatory role of ERR on energy metabolism in organisms is conservative. ERR also plays a critical regulatory role in regulating metabolic processes in insects, including glycolysis, carbohydrate metabolism, and lipid synthesis. For instance, ERR knockdown in *Drosophila* larvae decreased the expression of glycolysis-related genes, reduced ATP synthesis, and inhibited the catabolism of trehalose and glycogen, leading to larval death [10]. In *Drosophila* adults, reduced ERR expression disrupts the glycolytic pathway and fat synthesis [11]. Additionally, ERR regulates the glycolytic pathways in aphids and *Aedes aegypti*, with suppression of ERR expression in aphids downregulating the transcription of glycolysis-related genes, leading to a reduction in the number of progeny [12,13].

Although the transcriptional activity of vertebrate ERR is ligand-independent, it requires co-regulatory factors. In vertebrates, peroxisome proliferator-activated receptor gamma coactivator-1-related coactivator (PPRC1) is a member of the PGC-1 family of coactivators for ERRs [14] Coactivators activate the transcription of target genes by binding to the AF-2 structural domain of ERR, enabling it to recognize ERRE on the promoters of downstream target genes [3,15]. Since ERRs lack natural ligands, coactivators often function as “protein ligands” that bind to the AF-2 domain to facilitate transcriptional activation [16,17]. While ERR is an ancient nuclear receptor and its function and mode of action are evolutionarily conserved, the metabolic regulation and hormonal systems of insects differ significantly from those of vertebrates. Most insects have only one ERR; however, their functions and functional networks are complex. For instance, trehalose metabolism [18], the ecdysone signaling pathway [19–21], and the juvenile hormone signaling pathway, unique to insects, are regulated by ERR [22].

The AF-2 domain is a key region for ERR to recognize and bind coactivators to exert metabolic regulatory functions. However, the function of the ERR AF-2 structural domain in insects and whether cofactors are required for the ERR to exert its metabolic regulatory functions have not yet been clarified. Our previous studies demonstrated that the role of ERR in the silkworm (*Bombyx mori*; BmERR) in regulating glucose metabolism is similar to that in vertebrates. ERR is achieved by binding to an estrogen-related receptor response element (ERRE) on the promoters of target genes to regulate the expression of relevant genes [23]. BmERR regulates the embryonic development of silkworms by binding to ERRE on the promoters of glycolysis-related genes and regulating the expression of *trehalose* genes to maintain glucose homeostasis in the hemolymph [18,24].

This study analyzed the relationship between the AF-2 domain of insect ERR and its function. In *Drosophila*, however, ERR knockout is lethal [10], and partial deletion reduces survival [11], thereby greatly complicating functional analysis. Given its ease of rearing, high metabolic rate, and drug sensitivity, the silkworm serves as an ideal Lepidopteran model for research critical to advancing our understanding of metabolic mechanisms [25]. To achieve this, using the silkworm *B. mori* as a model, we knocked out the AF-2 domain of BmERR using CRISPR/Cas9 gene editing technology and examined the effects on its functional role, associated interacting factors, and related physiological processes.

## 2. Results

### 2.1 Generation of transgenic silkworms with BmERR AF-2 knockout

Analysis of the *BmERR* coding sequence revealed a suitable target site within the AF-2 domain (Fig 1A). Following CRISPR/Cas9-mediated editing, transgenic gRNA individuals with green fluorescence were successfully identified through fluorescence screening (Fig 1B). The positive transgenic gRNA silkworms were hybridized with transgenic nos-Cas9 silkworms to obtain the F1 generation. Sequencing analysis of genomic fragments from F1 individuals containing the gRNA target site revealed a 16-base pair deletion in the effective knockout form. This deletion led to abnormal encoding of the AF-2 domain in the BmERR protein, resulting in the premature termination of translation (Figs 1C, 1D and S1). Homozygous breeding was conducted on transgenic silkworms, successfully obtaining a transgenic mutant lacking the AF-2 domain of BmERR, designated as BmERR^∆AF-2^.

**Fig 1:**
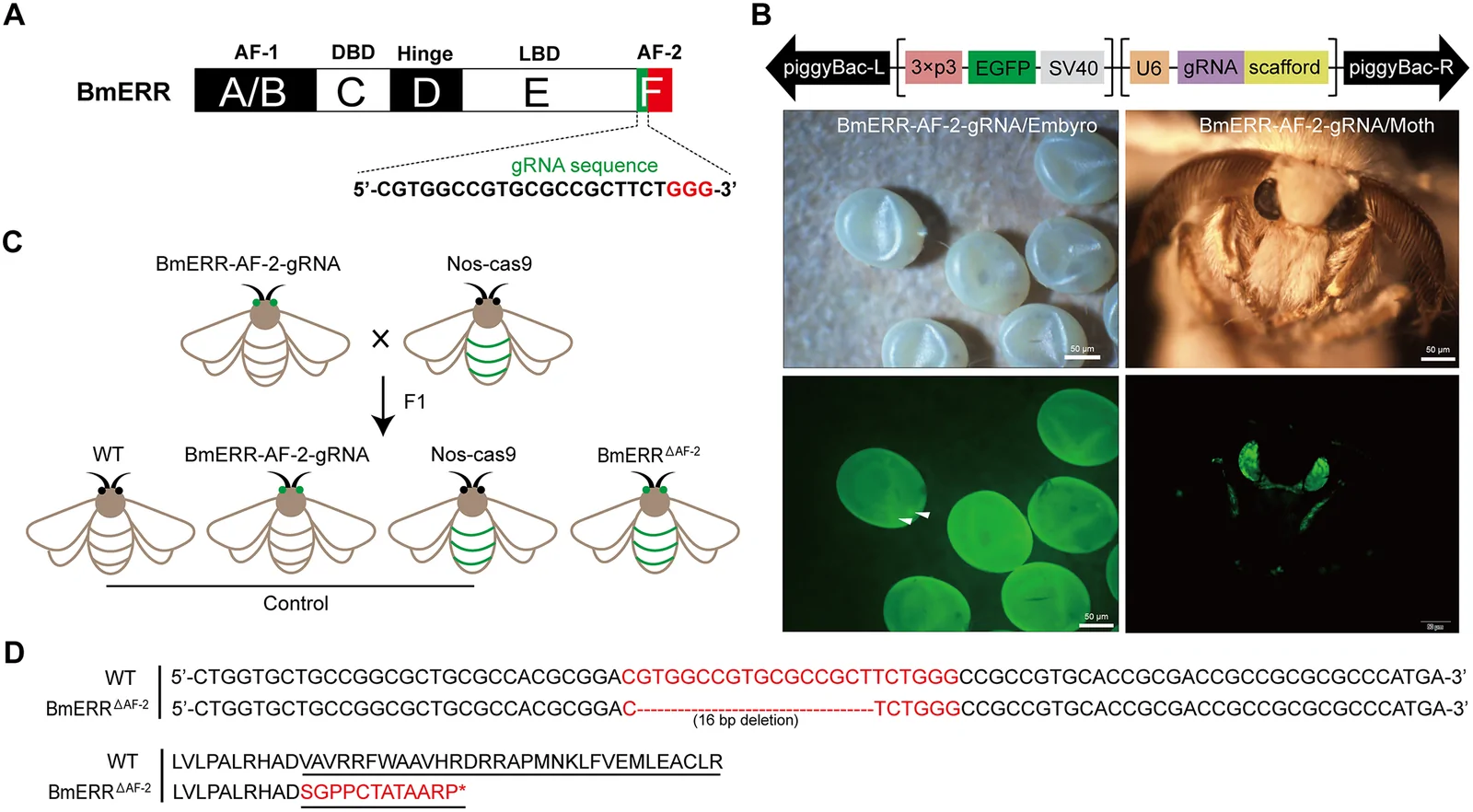
Transgenic silkworms with BmERR AF-2 knockout. **(A)** Schematic of the BmERR protein and the design of the gRNA target site. **(B)**: Screening process for transgenic gRNA silkworms. **(C)** Generation of transgenic silkworms with BmERR AF-2 domain knockout (BmERR^∆AF-2^). **(D)** Genotypic identification of the knockout silkworms. WT, wild type.

### 2.2 Knockdown of the BmERR AF-2 domain leads to the loss of epidermal marking in the silkworm

The phenotypes of transgenic knockout silkworms were observed. The results showed that compared to the controls, fourth-instar knockout larvae showed significantly lighter markings on the epidermis and fifth-instar knockout larvae lacked the eye-shaped, crescent, and star-shaped markings on the epidermis (Fig 2A). In addition, the absence of BmERR AF-2 domain also led to a decrease in body weight of silkworms (S2A Fig), a significant reduction in cocoon volume (S2B Fig), and a significant decrease in total cocoon weight, pupal weight, and cocoon layer rate, with no significant change in cocoon layer weight (S2C, S2D, S2E and S2F Fig).

**Fig 2:**
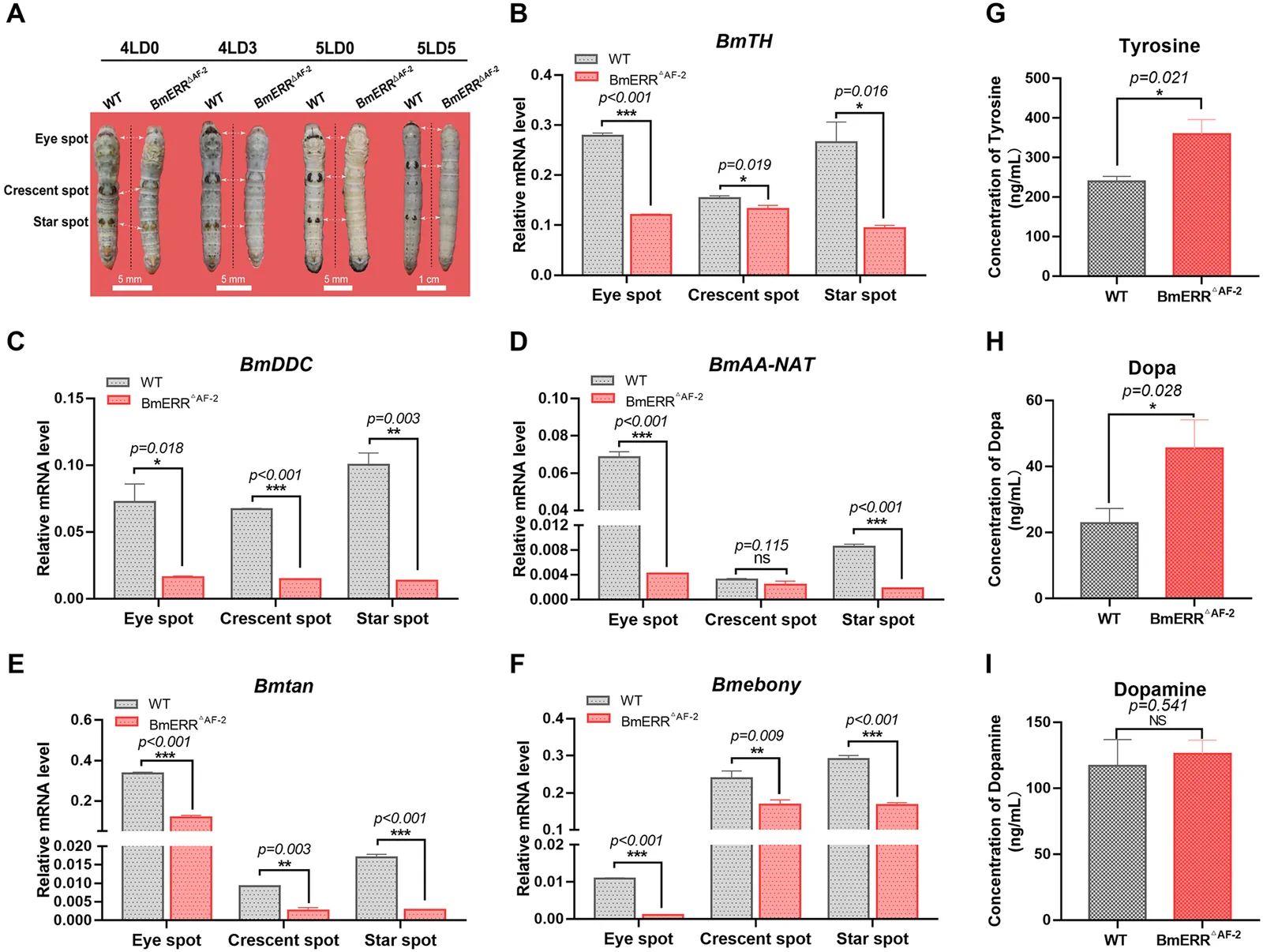
Deletion of the BmERR AF-2 domain inhibits melanin synthesis and leads to loss of epidermal markings in silkworms. **(A)** Epidermal phenotypes of wild-type (WT) and BmERR AF-2 knockout (BmERR^∆AF-2^) silkworms at the fourth and fifth instar stages. **(B-F)** Expression levels of melanin pathway-related genes—*BmTH* **(B)**, *BmDDC* **(C)**, *BmAA-NAT* **(D)**, *Bmtan* **(E)**, and *Bmebony* **(F)**—in the epidermis of WT and BmERR^∆AF-2^ silkworms during the fourth-instar stage. **(G-I)** Concentrations of melanin-related metabolites—Tyrosine **(G)**, Dopa **(H)**, and Dopamine **(I)**—in the epidermis of WT and BmERR^∆AF-2^ silkworms at the beginning of the fifth instar. Unit, ng/ml. Developmental stages are abbreviated as: 4LD0, beginning of the fourth instar; 4LD3, third day of the fourth instar; 5LD0, beginning of the fifth instar; 5LD5, fifth day of the fifth instar. The error bars represent mean ± SD (n = 3). Student’s t-test, *, p < 0.05; **, p < 0.01; ***, p < 0.001; NS, for p > 0.05. Each sample was measured in triplicate across three independent biological replicates.

The black markings on the insect epidermis are primarily formed by the deposition of melanin. The expression of genes related to the melanin metabolism pathway was examined in the marking regions on the epidermis of transgenic knockout silkworms at the fourth-instar molt stage. The results showed that *BmTH* and *BmDDC* were expressed in all three marking regions, and that the expression levels of *BmTH* and *BmDDC* in the marking regions of the transgenic knockout silkworm epidermis were significantly reduced (Fig 2B and 2C). *BmAA-NAT* and *Bmtan* were mainly expressed in the eye-shaped markings of the silkworm epidermis, whereas *Bmebony* was mainly expressed in the crescent and star-shaped markings. In the epidermal marking regions of the transgenic knockout silkworms, the expression levels of *BmAA-NAT* (Fig 2D), *Bmebony* (Fig 2F), and *Bmtan* (Fig 2E) were significantly reduced. The content of small-molecule metabolites in the melanin synthesis pathway in the epidermis of fifth-instar transgenic knockout silkworms after molting was detected. Compared with the controls, the contents of tyrosine (Fig 2G) and Dopa (Fig 2H) were significantly increased, whereas the dopamine (Fig 2I) content showed no significant change. This suggests that knocking out the AF-2 domain of BmERR suppresses the expression of genes related to the melanin metabolic pathway in the silkworm cuticle, thereby blocking melanin synthesis and leading to the disappearance of epidermal markings.

### 2.3 Overexpression of BmERR causes disordered epidermal markings in silkworms

To verify the regulatory role of the BmERR AF-2 domain in the formation of silkworm epidermal markings, we constructed a transgenic vector for the systemic overexpression of BmERR. After microinjecting the silkworm eggs, we screened for individuals with red fluorescence in their eyes and obtained transgenic silkworms overexpressing BmERR (Fig 3A). Epidermal markings were observed in transgenic overexpression silkworms. The body markings of the fourth- and fifth-instar silkworms overexpressing BmERR were significantly different from those of the control silkworms. The eye-shaped, crescent, and star-shaped markings were much darker in color, and black markings also appeared in the intersegmental membrane regions of each body segment (Fig 3B). During the second instar to the cocooning period, the expression levels of the *BmERR* gene (Fig 3C) and BmERR protein (Fig 3D) in the epidermis of transgenic overexpressing silkworms were significantly higher than those in the wild-type.

**Fig 3:**
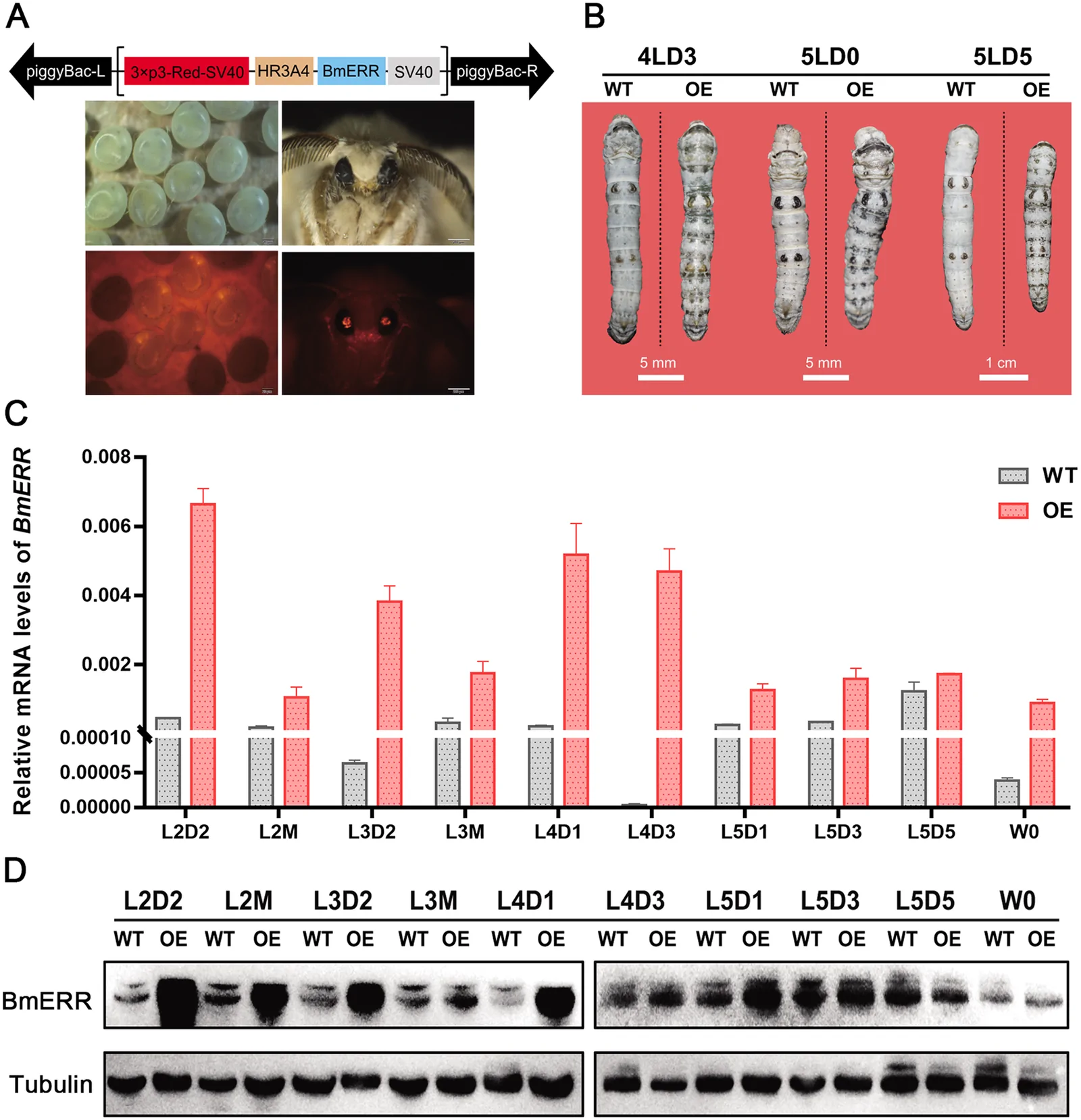
Overexpression of BmERR enhances epidermal markings in silkworms. **(A)** Generation of BmERR-overexpressing transgenic silkworms. **(B)** Epidermal phenotypes of transgenic BmERR-overexpressing (OE) silkworms at different developmental stages. **(C)** *BmERR* transcript levels in the epidermis of OE silkworms. **(D)** BmERR protein expression in the epidermis of OE silkworms. Developmental stages are abbreviated as follows: L2D2, second day of the second instar; L2M, second instar molt; L3D2, second day of the third instar; L3M, third instar molt; L4D1 and L4D3, first and third days of the fourth instar; L5D1, L5D3, and L5D5, first, third, and fifth days of the fifth instar; W0, cocooning stage. WT: wild type silkworm. The error bars represent mean ± SD (n = 3). Student’s t-test, *, p < 0.05; **, p < 0.01; ***, p < 0.001; NS for p > 0.05. Each sample was measured in triplicate across three independent biological replicates.

The expression of genes related to the melanin metabolism pathway was detected in the epidermis of fourth-instar transgenic silkworms overexpressing BmERR. The expression levels of *BmTH* and *BmDDC* were significantly increased (Fig 4A and 4B), as were the expression levels of *BmAA-NAT*, *Bmtan*, and *Bmebony* (Fig 4C and 4E). In addition, the expression levels of transcription factor Bmapt-like and Toll pathway ligand Bmspz3 were significantly reduced in the epidermis of BmERR^∆AF-2^ silkworms (S3A and S3B Fig) but significantly increased in the epidermis of silkworms overexpressing BmERR (S3C and S3D Fig). These changes in gene expression indicate that the increased expression of BmERR in the silkworm cuticle upregulates the melanin metabolic pathway, ultimately promoting melanin synthesis and deposition in the cuticle, thereby forming black pigmentation.

**Fig 4:**
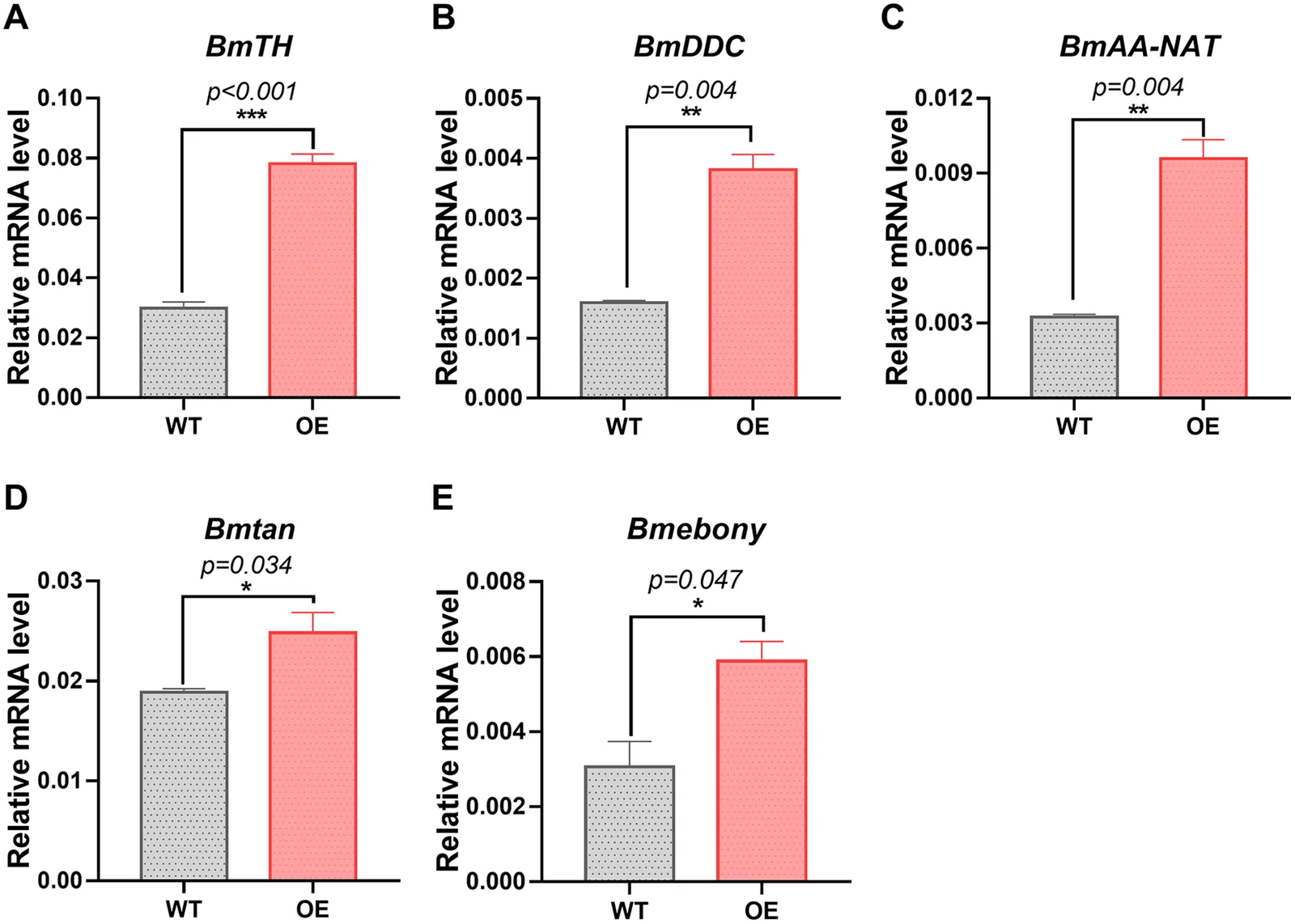
Expression analysis of melanin metabolism pathway genes in the epidermis of BmERR-overexpressing silkworms. **(A–E)** Expression levels of key melanogenic genes—*BmTH* **(A)**, *BmDDC* **(B)**, *BmAA-NAT* **(C)**, *Bmtan* **(D)**, and *Bmebony* **(E)**—in the epidermis of wild-type (WT) and BmERR-overexpressing (OE) silkworms at the fourth-instar molt stage. The error bars represent mean ± SD (n = 3). Student’s t-test, *, p < 0.05; **, p < 0.01; ***, p < 0.001; NS for p > 0.05. Each sample was measured in triplicate across three independent biological replicates.

### 2.4 BmERR and BmPPRC1 synergistically regulate the expression of genes related to epidermal markings formation

Vertebrate ERRs require the involvement of coactivators to perform metabolic regulatory functions, and the AF-2 structural domain is the region where ERRs recognize and bind coactivators. PPRC1, a member of the PGC-1s family, is an important metabolic regulatory coactivator of ERR [14]. We identified the PPRC1 homologous gene (*BmPPRC1*) in silkworms. Evolutionary analysis revealed that *BmPPRC1* has a closer genetic relationship with *Plutella xylostella* and *Pecctinophora gossypiella* (S4 Fig). To investigate whether BmPPRC1 is a coactivator of BmERR, we examined the expression of BmPPRC1 and BmERR in the epidermis of the second instar dormant to wandering silkworms. The assay results showed that the expression trends of BmERR and BmPPRC1 were very similar in the epidermis at the larval stage (Fig 5A). We then co-overexpressed BmERR and BmPPRC1 in the BmE cells. The cellular immunofluorescence results showed that both BmERR and BmPPRC1 were localized in the nucleus and that the positions of the fluorescent signals largely overlapped (Fig 5B). We then co-transfected BmERR and BmERRM (BmERR lacking the AF-2 structural domain) with BmPPRC1 in BmE cells and detected their binding using the co-immunoprecipitation assay. This assay showed that BmERR bound to BmPPRC1 through its AF-2 structural domain. When the AF-2 structural domain was deleted, BmERR lost its ability to bind BmPPRC1 (Fig 5C).

**Fig 5:**
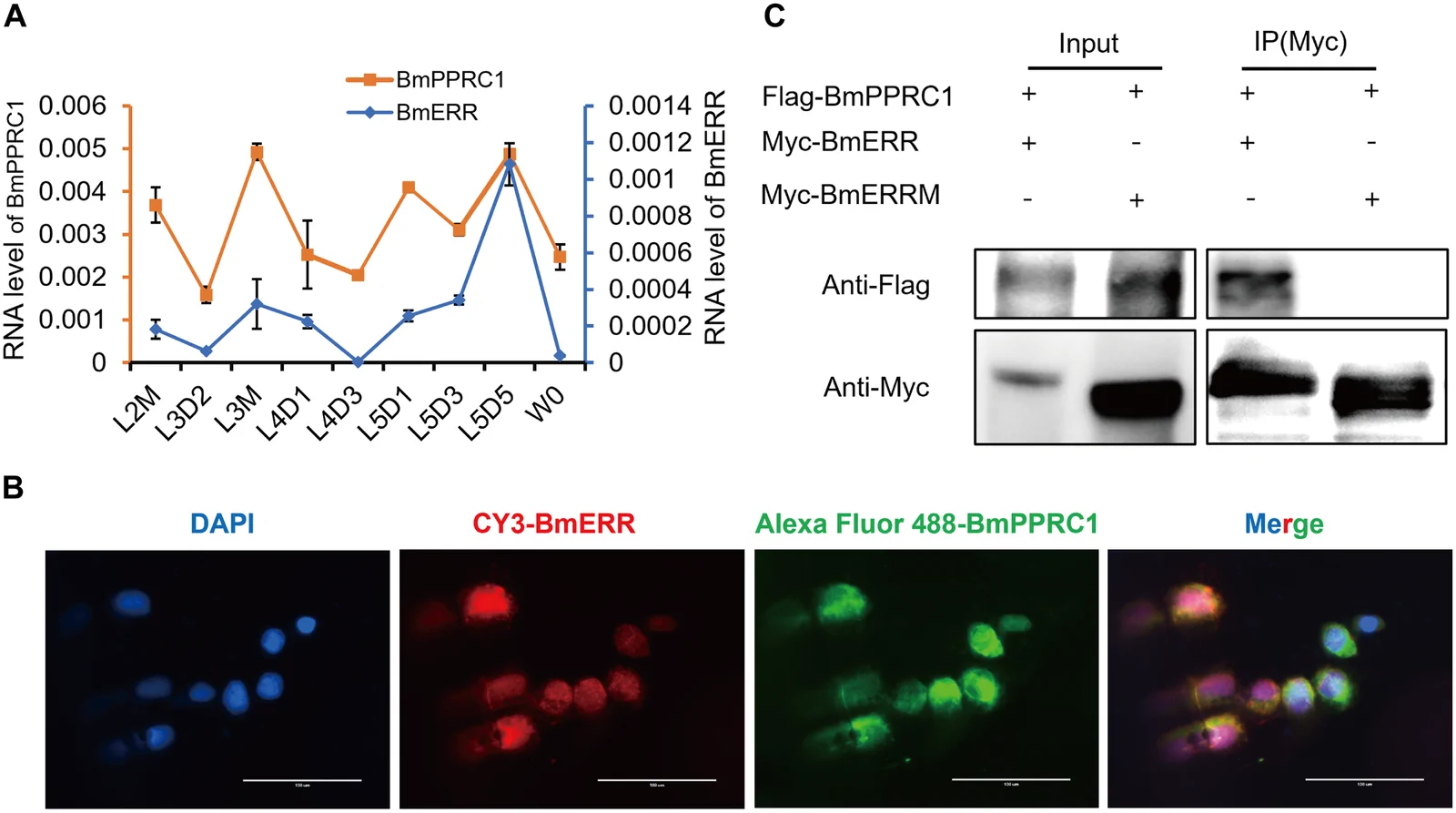
Interaction between BmERR and BmPPRC1. **(A)** Expression patterns of *BmPPRC1* and *BmERR* in the silkworm epidermis across developmental stages. **(B)** Subcellular co-localization of BmPPRC1 and BmERR in BmE cells. Scale bar, 100 μm. **(C)** Co-immunoprecipitation (Co-IP) assay confirming the physical interaction between BmPPRC1 and BmERR. The error bars represent mean ± SD (n = 3). Student’s t-test, *, p < 0.05; **, p < 0.01; ***, p < 0.001; NS for p > 0.05. Each sample was measured in triplicate across three independent biological replicates.

BmERR and BmERRM were incrementally coexpressed with BmPPRC1 in BmE cells. The results revealed that truncation of the AF-2 structural domain of BmERR resulted in a significant reduction in the expression of the melanin metabolism pathway-related genes *Bmebony* and *Bmlac2*, as well as marking formation-related regulators *Bmspz3* and *Bmapt-like* (Fig 6A). Analysis of the nucleic acid sequences of the promoter regions 2000 bp upstream of the transcription start sites of these genes revealed the presence of one or more potential ERRE-like CRE in the regions upstream of the transcription start sites of *Bmebony*, *Bmtan*, *Bmspz3*, and *Bmapt-like* genes (S5A Fig). Subsequently, BmERR and BmPPRC1 were overexpressed separately in BmE cells, and nuclear proteins were extracted for EMSA to verify whether BmPPRC1 could facilitate the binding of BmERR to ERRE like elements. EMSA results demonstrated that the addition of BmPPRC1 strengthened the capacity of BmERR to bind ERRE like elements on the target gene promoters (S6 Fig).

**Fig 6:**
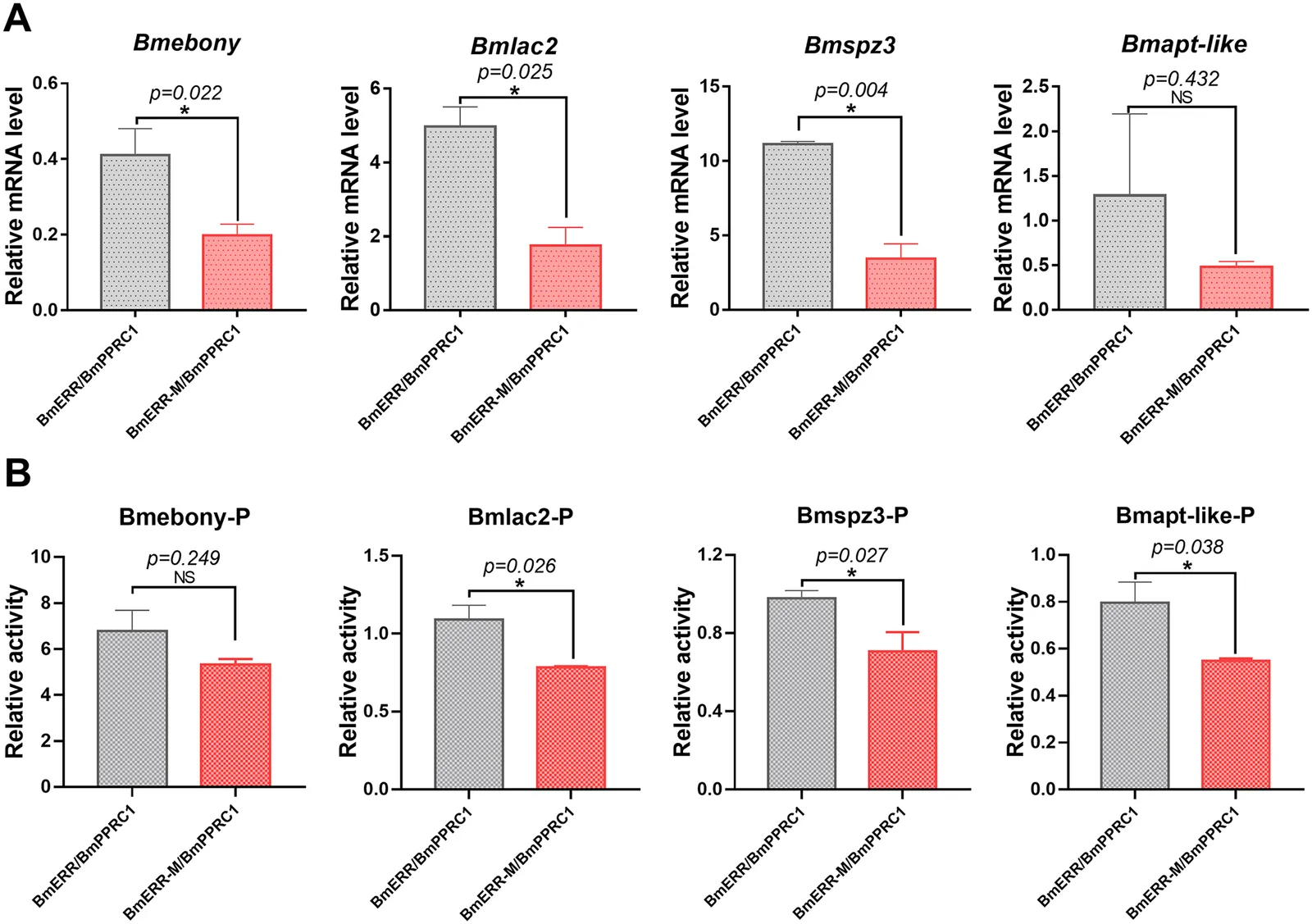
BmERR regulates speckle formation-related gene expression through the AF-2 domain and BmPPRC1. **(A)** Expression levels of *Bmebony*, *Bmlac2*, *Bmspz3*, and *Bmapt-li*ke in BmE cells following overexpression of BmPPRC1 and the AF-2 domain-truncated BmERR (BmERR^∆AF-2^). **(B)** Transcriptional activity of the corresponding gene promoters under the same experimental conditions. The error bars represent mean ± SD (n = 3). Student’s t-test, *, p < 0.05; **, p < 0.01; ***, p < 0.001; NS for p > 0.05. Each sample was measured in triplicate across three independent biological replicates.

Nucleic acid sequences of the promoter region 2000 bp upstream of the transcription start site of the melanin metabolism pathway-related genes *Bmebony* and *Bmlac2*, as well as marking formation-related regulators *Bmspz3* and *Bmapt-like* genes, were cloned, and cellular transfection vectors driving luciferase expression were constructed. Forty-eight hours after the transfection of BmE cells, luciferase activity assays showed that the core promoters of these genes were transcriptionally active (S5B Fig). Upon co expression of BmERRM and BmPPRC1, the promoter activities of *Bmebony*, *Bmlac2*, *Bmspz3* and *Bmapt-like* were markedly lower than those observed under co-expression of BmERR and BmPPRC1 in BmE cells (Fig 6B). These results indicate that BmERR regulates the expression of genes related to pigmentation formation by binding to BmPPRC1 through its AF-2 domain.

## 3. Discussion

ERRs are orphan nuclear receptors that are constitutively active in vertebrates and can recognize and bind coactivators to synergize their transcriptional regulatory functions in the absence of ligands via AF-2 [26]. Deletion of the AF-2 functional domain of the aphid ERR affects its nuclear localization, leading to translocation of the aphid ERR into the cytoplasm [13]. In the present study, the knockdown of the BmERR AF-2 structural domain resulted in the disappearance of black markings on the epidermis of silkworms, whereas overexpression of BmERR increased the black markings on the epidermis of silkworms. This suggests that the AF-2 structural domain of insect ERR is functionally important and may be involved in the formation of insect epidermal markings, which has not been previously reported in insect ERR studies. Notably, while the complete knockout of *Drosophila* ERR is lethal [10], BmERR^∆AF-2^ knockout silkworms remained but had significantly lower body weight and cocoon economic traits at the fifth instar. This implies that the knockout of the BmERR AF-2 domain did not cause lethality in silkworms, despite its negative effects on growth, development, and energy metabolism.

Deletion of the structural domain of BmERR AF-2 resulted in the loss of markings on the silkworm epidermis. Black markings of the insect epidermis are mainly formed by melanin deposition [27]. Tyrosine hydroxylase (TH) and dopamine decarboxylase (DDC) are key enzymes involved in melanin synthesis. Aberrant expression of TH and DDC in *Drosophila*, *Tribolium castaneum*, *Platymeris biguttatus*, *Precis coenia*, and *Pseudaletia separata* leads to abnormal body color or markings [28–32]. Significant reductions in *BmTH* and *BmDDC* expression were observed in the epidermal marking regions of the transgenic knockout silkworms. Dopa and dopamine are precursors of insect melanin, which can be catalyzed by phenol oxidases to form dopa melanin and dopamine melanin, respectively, which are deposited in the insect epidermis to form black markings and body coloration [33–35]. Tyrosine and Dopa levels were significantly higher in the epidermis of transgenic knockout silkworms than in the epidermis of wild-type silkworms, indicating that the absence of the AF-2 domain of BmERR in the fourth instar silkworms leads to reduced expression of *BmTH* and *BmDDC* in the melanin metabolism pathway. This inhibition reduces the synthesis of dopa melanin and dopamine melanin, leading to the accumulation of tyrosine and dopa in the epidermis of the fifth instar transgenic knockout silkworms and resulting in the loss of their epidermal markings. In contrast, the overexpression of BmERR in the epidermis of silkworms upregulated the expression of *BmTH* and *BmDDC* and promoted melanin synthesis.

The dopamine branching metabolic pathway involves key genes such as AA-NAT, ebony, and tan. Dopamine can either be incorporated into dopamelanin or serve as a substrate for Bmebony and BmAA-NAT to produce N-β-alanyl dopamine (NBAD) and N-acetyl dopamine (NADA), respectively [27,36]. Consistently, the deletion of the AF-2 domain suppressed the expression of *BmAA-NAT*, *Bmebony*, and *Bmtan*, whereas overexpression of BmERR enhanced their transcription. Toll pathway ligand *Bmspz3* regulates epidermal melanin deposition in silkworms by binding to the Toll-8 receptor [37]. The transcription factor *Bmapt-like* also serves as a key regulator in the formation of epidermal markings in silkworms [38]. In this study, the expression levels of *Bmapt-like* and *Bmspz3* were significantly reduced in transgenic knockout silkworms and significantly elevated in transgenic overexpressing silkworms. These results indicate that BmERR may regulate silkworm epidermal marking formation by regulating the expression of marking-related transcription factors.

The AF-2 structural domain is a key site for ERR to recognize and bind coactivators. In vertebrates, PGC-1 is an important coactivator of ERR and plays a synergistic regulatory role in mitochondrial biogenesis and bioenergetic regulation [14,17,39,40]. In this study, the expression trend of *BmPPRC1*, a homologue of the ERR coactivator PGC-1s in silkworm, in the epidermis was very similar to that of BmERR. In addition, its fluorescence localization signals in the cells largely overlapped with those of BmERR. We confirmed that BmERR binds to BmPPRC1 through the AF-2 structural domain through co-immunoprecipitation experiments. BmERR is required for transcriptional regulation by binding to ERRE in the promoters of downstream target genes. Analysis of the promoter sequences of the melanin metabolism pathway genes and *Bmapt-like* and *Bmspz3* genes of the silkworm revealed the presence of BmERR-binding elements in the 2-kb region upstream of the *Bmebony, Bmlac2, Bmapt-like, and Bmspz3* genes. The results of the gene expression and luciferase enzyme activity assays indicated that the AF-2 structural domain affects the regulation of gene expression by BmERR and BmPPRC1, which are involved in the formation of epidermal markings in silkworms.

The absence of the ERR AF-2 domain leads to the loss of epidermal markings in the silkworm. However, as a domesticated insect primarily reared under artificial conditions, this loss has minimal impact on its growth and development. In other insects, melanization holds significant physiological importance, playing crucial roles in UV resistance, adaptation to low-temperature environments, and immune regulation. For instance, the black or dark brown markings on aphids provide protective effects against UV damage [41]. Similarly, melanization in the cuticle of *Agelaia pallipes* and *Oncopeltus fasciatus* enhances heat absorption and offers photoprotection, improving their resistance to ultraviolet radiation and cold environments [42,43]. In *Allonemobius socius*, darker individuals exhibit higher phenoloxidase activity and greater resistance to bacteria [44]. These studies collectively indicate that the loss of melanin can adversely affect insect health. ERR functions as a stress-response factor in insects and can be induced by stressors such as UV exposure and low temperatures [45]. Therefore, it remains to be further validated whether ERR influences epidermal pattern formation in these insects by regulating melanin synthesis, thereby aiding in resistance to external stressors.

Despite significant metabolic differences between insects and vertebrates, the function of ERR is evolutionarily conserved. Our results demonstrate that, similar to its vertebrate counterpart, BmERR functions as a transcriptional regulator by interacting with the coactivator BmPPRC1 via its AF-2 domain. Furthermore, insect integumentary patterning is intrinsically linked to pigment metabolism. We discovered that BmERR influences the formation of these epidermal markings by participating the melanin pathway. Regrettably, in this study, the systemic knockout of the BmERR AF-2 domain suggests that the changes in epidermal markings may also be caused by metabolic disorders in other tissues (such as fat body), indirectly affecting melanin synthesis, rather than being a direct result of the absence of the BmERR AF-2 domain in the epidermis.

## 4. Conclusion

Through its AF-2 domain, BmERR binds to BmPPRC1 to coregulate the expression of genes in the melanin metabolic pathway. This coordinated regulation controls melanin synthesis and ultimately influences the formation of epidermal markings in silkworms. This study uncovers a novel role for ERR in regulating insect marking formation, thereby challenging the established paradigm that confines its function to energy metabolism. Thus, our study not only confirms the conservation of the ERR regulatory mechanism but also reveals its diverse functional roles.

## 5. Materials and methods

### 5.1 Insects

The D9L and Nos-cas9 transgenic silkworm lines were provided and maintained by the Biological Science Research Center, Southwest University, Chongqing. The larvae were fed with fresh mulberry leaves daily under controlled conditions of 26 ± 1°C temperature, 80 ± 10% relative humidity, and a 12-h light/12-h dark photoperiod.

### 5.2 gRNA design and construction of transgenic knockout vectors

The CCtop tool [46] was used to design BmERR AF-2 structural domain-specific gRNA. The protospacer-adjacent motifs (PAMS) are an essential component of the CRISPR-Cas9 genome editing system, typically 2–5 base pairs in length, located adjacent to protospacers in CRISPR loci, which can guide the Cas9 protein to perform targeted cleavage of target sequences [47]. GGG was used as the PAM in this study. The recognition sequence of Aar Ⅰ restriction endonuclease was added at both ends of the AF-2 gRNA sequence, synthesized as two reverse complementary oligos (Sangon Biotech, Shanghai, China), annealed at 95°C for 5 min, and inserted into the Aar Ⅰ digested piggyBac [3 × p3-EGFP-SV40, U6-scaffold] backbone to construct piggyBac [3 × p3-EGFP-SV40, U6-gRNA-scaffold] vector.

### 5.3 Preparation of transgenic silkworms

The *piggy*Bac [3 × p3-EGFP-SV40, U6-gRNA-scaffold] vector was mixed with the helper plasmid in a 1:1 ratio of substance (400 ng/μL) and subsequently microinjected into D9L silkworm eggs. After hatching, the G0 generation silkworms were reared until they developed into moths, after which same-batch mating was performed to produce G1 generation individuals. After 6 days of normal development, the G1 eggs were placed under a fluorescence microscope to observe their fluorescence. Eggs with green, fluorescent eyes were screened as positive for G1 generation transgenic gRNA silkworms. Transgenic gRNA silkworm individuals were crossed with Nos-cas9 silkworm lines to obtain F1 generation individuals. The Cas9 protein in transgenic nos-Cas9 silkworms is regulated by the Nanos promoter and expressed throughout the body [48]. Under a fluorescence microscope, BmERR AF-2 knockout silkworms were identified as those with green fluorescence in both eyes and the body in the F1 generation, those with green fluorescence only in the eyes were identified as transgenic AF-2 gRNA silkworms, those with green fluorescence only in the body were identified as Nos-cas9 silkworms, and those without green fluorescence were identified as normal silkworms. The previously constructed *piggy*Bac [3 × p3-Red-SV40, HR3A4-BmERR-SV40] vector [18] was microinjected into the D9L silkworm eggs in the same manner. Transgenic silkworms overexpressing BmERR were obtained using fluorescence screening to obtain silkworms with eye-specific red fluorescence.

### 5.4 Phenotypic statistics

Based on previously reported methods [49], the body weight at the fifth instar and the economic traits of cocoons were measured in transgenic knockout silkworms. Silkworms were weighed at the fifth instar stage, and the average weight per silkworm was calculated (n = 30). The cocoon weight, cocoon shell weight, and pupal weight of wild-type silkworms and transgenic silkworms were determined on the third day of the pupal stage, respectively (n = 30).

### 5.5 Genome extraction and genome sequencing

The epidermis of transgenic knockout silkworm larvae was collected on the third day of the fifth instar, and genomic DNA was extracted using a Tissue DNA kit (OMEGA, Norcross, GA, USA). Target sites in the genomic DNA were amplified by PCR and sequenced. The primer sequences are listed in S1 Table.

### 5.6 RNA extraction and quantitative real-time PCR validation

TRIzol reagent (Invitrogen, Waltham, MA, USA) was used to extract total RNA from the epidermis of transgenic knockout silkworms and transgenic silkworms overexpressing different instars. Total RNA was digested with *DNase I* (TaKaRa Biotech, Kyoto, Japan) to avoid genomic DNA contamination and then reverse-transcribed into cDNA using the M-MLV Reverse Transcription Kit (Promega, Madison, WI, USA) and stored at -20°C. Quantitative real-time PCR was performed using NovoStart SYBR qPCR SuperMix Plus (Novoprotein, Suzhou, China) and an ABI StepOne v2.1 Sequence Detection System (Applied Biosystems, Foster City, CA, USA). Relative quantification of target gene expression was determined through the 2^−ΔΔCt^ method, the silkworm translation initiation factor 4A (*BmTIF4A*) was used as an endogenous control. The PCR amplification program included an initial denaturation step at 95°C for 60 s, followed by 40 cycles of denaturation at 95°C for 20 s, extension at 60°C for 60 s, and extension at 72°C for 35s. Primers used are listed in S1 Table.

### 5.7 Western blotting

The silkworm tissue material was quickly frozen in liquid nitrogen, ground into a powder in a mortar, and transferred to a 1.5-mL centrifuge tube. RIPA lysis buffer (500 μL; Beyotime Biotechnology, Shanghai, China) and an appropriate amount of protease inhibitors were added to the centrifuge tube. After thorough mixing, the mixture was placed on ice for 30 min, followed by centrifugation at 13000 × g for 15 min at 4°C. The supernatants were collected.

The total protein concentration was determined using a BCA Protein Assay Kit (Beyotime Biotechnology). A sample of 100 μg was subjected to sodium dodecyl sulfate polyacrylamide gel electrophoresis on a 10% polyacrylamide gel, followed by transfer of the proteins to a PVDF membrane (Roche, Basel, Switzerland). Membranes were probed with antibodies against BmERR (BmERR antibody was stored in our laboratory [50] and tubulin (endogenous control, Beyotime Biotechnology, Shanghai, China). Horseradish peroxidase-conjugated goat anti-rabbit IgG (Beyotime Biotechnology) was used as the secondary antibody. Enhanced chemiluminescence was used to detect immune complexes and protein blots were imaged using a Clinx ChemiScope 3400 Mini machine (Clinx Science Instruments Co., Shanghai, China).

### 5.8 Determination of tyrosine, dopa, and dopamine

Standard solutions were prepared by weighing an appropriate amount of the standard substances of the three compounds (tyrosine, dopa, and dopamine) into 10% methanolic formic acid. Then, appropriate volumes of each standard solution were measured to create a mixed standard solution, which was diluted stepwise with 10% methanolic formic acid to the desired concentration to obtain working standard solutions.

For extraction, 50 mg of silkworm epidermis was mixed with 600 μL of 10% methanolic formic acid-H_2_O (1:1, V/V) solution in a 2 mL EP tube. Then, two steel beads were added, vortexed for 30 s, ground in a tissue grinder at 60 Hz for 90 s, and centrifuged at 12000 rpm for 5 min at 4°C. Then, 100 μL of supernatant was mixed with 100 μL of isotopic internal standard solution (concentration of 100 ppb) and vortexed for 30 s. The supernatant was filtered through a 0.22-μm membrane and the filtrate was transferred to a vial for low-concentration substance detection. Then, 190 μL of 10% methanolic formic acid-H_2_O (1:1, V/V) solution was added to 10 μL of the original supernatant and vortexed for 30 s. Next, 100 μL of the diluted sample was vortexed with 100 μL of isotopic internal standard solution (concentration of 100 ppb) for 30 s. The supernatant was filtered through a 0.22 μm membrane and transferred to a vial for high-concentration substance detection. Quantitative analyses and calculations were performed using liquid chromatography-mass spectrometry.

The chromatographic conditions were as follows: Column: ACQUITY UPLC BEH C18 column (2.1 × 100 mm, 1.7 μm, Waters Corporation, Milford, Massachusetts, USA). Injection volume: 5 μL. Column temperature: 40°C. Mobile phase A: 10% methanol in water (with 0.1% formic acid); mobile phase **(B)** 50% methanol in water (with 0.1% formic acid). Gradient elution conditions: 0–6.5 min, 10–30% B; 6.5–7 min, 30–100% B; 7–14 min, 100% B; 14–17.5 min, 100–10% B. Flow rate: 0–8.0 min, 0.3 mL/min; 8.0–17.5 min, 0.4 mL/min.

Mass spectrometry conditions included an electrospray ionization source and positive ion mode: ion source temperature 500°C; ion source voltage, 5500 V; collision gas, 6 psi; curtain gas, 30 psi; nebulizer gas and auxiliary gas, 50 psi. Multiple reaction monitoring was performed for scanning.

### 5.9 Cell transfection and luciferase assays

Using the high-fidelity DNA polymerase (TransGen Biotech, Beijing, China), the promoter fragments of *Bmebony, Bmlac2, Bmspz3, and Bmapt-like* were cloned from the silkworm genomic DNA with different primers (S1 Table) under the following program: 95°C for 5 min; 35 cycles of 95°C for 30 s, 56°C for 30 s, 72°C for 2 min, and 72°C for 10 min. The promoter fragments were then inserted into the firefly luciferase reporter vector pGL3-Basic (Promega).

A luminescent reporter assay was performed according to the manufacturer’s instructions. The BmE cell line used in this study was originally established from silkworm embryonic cells and has been maintained in our laboratory. The cells utilized in the experiments had undergone six passages. BmE cells were cultured in Grace insect cell culture medium (Gibco, Waltham, MA, USA) containing 10% fetal bovine serum (Gibco) in 24-well plates at 27°C. After 12 h, every well was transfected with a mixture of 1 μg recombinant plasmid, 0.1 μg internal control plasmid pRL-78ML [51], and 3 μL of Lipofectamine 2000 (Invitrogen) in insect medium without fetal bovine serum (FBS). After 6 h, the transfection mixture was replaced with 500 μL fresh insect medium containing 10% FBS. The cells were harvested 48 h after transfection and assayed using a Dual-Luciferase Reporter System (Promega).

### 5.10 Co-immunoprecipitation

To identify a potential coactivator for BmERR, we screened the silkworm gene database (https://silkdb.bioinfotoolkits.net/main/species-info/-1) to obtain a homologous gene of the Peroxisome proliferator-activated receptor gamma coactivator (PGC-1) family, PPRC1, referred to as *BmPPRC1*.

The psl 1180-HR3-A4-DsRed-SV40 vector was stored in our laboratory and overexpressed in *B. mori* embryonic (BmE) cells. Using the ligation-free cloning kit (Abm, Zhenjiang, China), the open reading frame of *BmPPRC1* was amplified from the cDNA and cloned into the psl 1180-HR3-A4-SV40 vector restricted with *BamH I* and *Not I* for homologous recombination. Primers used are listed in S1 Table.

Forty-eight hours after transfection, the cells were collected and lysed on ice for 10 min in NP-40 lysis buffer (Beyotime Biotechnology) containing 1 mM protease inhibitor mixture (Beyotime Biotechnology). After low-temperature centrifugation, the supernatant was collected, and antibody protein complexes were obtained according to the manufacturer’s instructions for the co-immunoprecipitation kit (Vazyme Biotech, Nanjing, China). Finally, the eluted samples were subjected to Western blotting.

### 5.11 EMSA

Probes for electrophoretic mobility shift assay (EMSA) were synthesized by Sangon Biotech (China) based on the nucleotide sequences of predicted ERRE‑like motifs in the promoters of *Bmebony*, *Bmlac2*, *Bmspz3*, and *Bmapt‑like*. The probe sequences are listed in S1 Table. To assess the interactions between these regulatory elements and overexpressed nuclear proteins BmERR and BmPPRC1, EMSA was carried out using a Chemiluminescent EMSA Kit (Beyotime, China). Reaction mixtures were incubated at 25 °C for 40 min, then loaded onto 5% native polyacrylamide gels for electrophoresis in Tris‑borate‑EDTA buffer (45 mM Tris‑borate, 1 mM EDTA; pH 8.3). Separated proteins were transferred onto a nylon membrane (Roche, USA). Following incubation with streptavidin‑horseradish peroxidase, chemiluminescent signals were detected using a Clinx ChemiScope 3400 Mini imaging system (Science Instruments, China).

### 5.12 Statistical analysis

The mean ± standard error was compared with the following significance thresholds using the Student’s t-test: *, p < 0.05, **, p < 0.01, and ***, p < 0.001. All statistical analyses were performed using Microsoft Excel (Microsoft Corp., Redmond, WA, USA). Graphs were drawn using Adobe Photoshop 2022 and Adobe Illustrator 2022.

## Supporting information

S1 Fig Identification of transgenic silkworms with BmERR AF-2 domain knockout. **(A)** Sequencing chromatograms of the gRNA target sites in genomic DNA from wild-type (WT) and BmERR AF-2 knockout (BmERR^∆AF-2^) silkworms. **(B)** Schematic diagram of the knockout sequence of the AF-2 domain of BmERR. **(C)** Western blot analysis of BmERR protein expression in the epidermal tissue of fifth-instar larvae from WT and BmERR^∆AF-2^ strains.
(TIF)

S2 Fig Knocking out the BmERR AF-2 domain affects the weight and cocoon economic traits of silkworms. **(A)** Body weight of transgenic knockout silkworms at the fifth instar. **(B)** Cocoon volume analysis of transgenic knockout silkworms. **(C-F)**: Statistical analysis of cocoon economic traits on the third day of the pupal stage: **(C)** cocoon weight, **(D)** pupal weight, **(E)** cocoon shell weight, and **(F)** cocoon shell ratio. The error bars represent mean ± SD (n = 3). Student’s t-test, *, p < 0.05; **, p < 0.01; ***, p < 0.001; NS for p > 0.05. Each sample was measured in triplicate across three independent biological replicates.
(TIF)

S3 Fig Expression of *Bmspz3* and *Bmapt-like* in the epidermis of transgenic silkworms. **(A)** and **(B)** Expression levels of *Bmspz3* **(A)** and *Bmapt*-*like* **(B)** in the epidermis of wild-type (WT) and BmERR AF-2 knockout (BmERR^∆AF-2^) silkworms. **(C** and **D):** Expression levels of *Bmspz3* (**C**) and *Bmapt*-*like* **(D)** in the epidermis of WT and BmERR-overexpressing (OE) silkworms. The error bars represent mean ± SD (n = 3). Student’s *t*-test, *, p < 0.05; **, p < 0.01; ***, p < 0.001; NS for p > 0.05. Each sample was measured in triplicate across three independent biological replicates.
(TIF)

S4 Fig Evolutionary analysis of the *PPRC1* gene in insects. (TIF)

S5 Fig Analysis of melanin metabolism-related gene promoters. **(A)** Schematic representation of predicted ERRE-like elements in the promoters of key melanin metabolism-related genes. **(B)** Luciferase reporter assays evaluating the transcriptional activity of the corresponding gene promoters. The error bars represent mean ± SD (n = 3). Student’s *t*-test, *, p < 0.05; **, p < 0.01; ***, p < 0.001; NS for p > 0.05. Each sample was measured in triplicate across three independent biological replicates.
(TIF)

S6 Fig BmPPRC1 promotes the binding of BmERR to ERRE core response elements (CRE) on the promoters of the *Bmebony*, *Bmlac2*, *Bmspz3*, and *Bmapt-like* genes. (TIF)

S1 Table Primers used for this study. (DOCX)

S1 Rawgel Uncropped and unprocessed raw images of all western blots (Figs 3D, 5C, S1C and S6S) and original photographic records (Figs 2A, 3B and S2B) presented in this study. (PDF)
