# Supplementary material for: The AF-2 domain of estrogen-related receptor interacts with PPRC1 to modulate larval epidermal marking in Bombyx mori
Source: PLoS Genet. 2026 Oct 12;22(10):e1012339. doi: 10.1371/journal.pgen.1012339 (PMC13657282; doi:10.1371/journal.pgen.1012339)

Raw images--blot

Figure 3D

Chemiluminescence detection

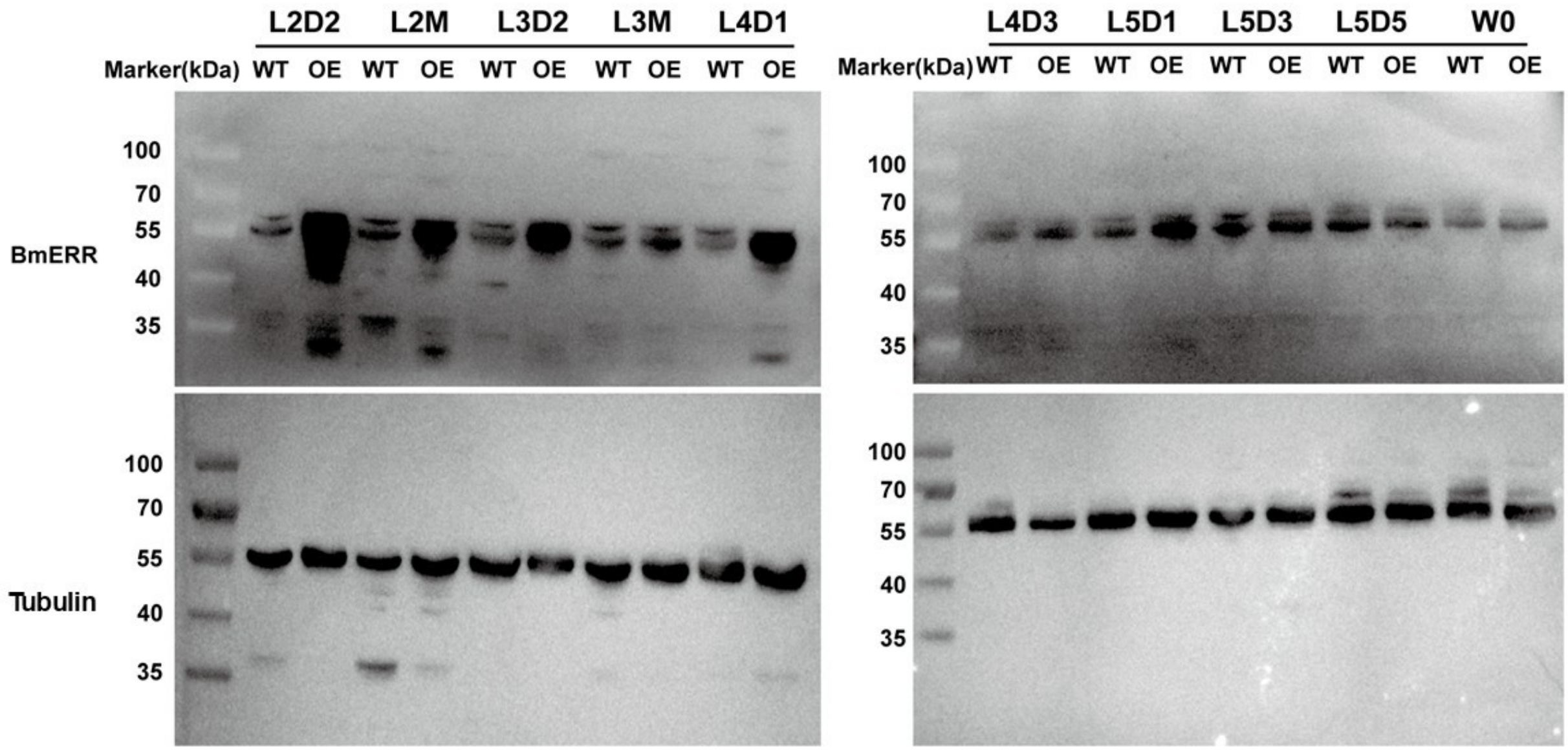

Figure 5C (upper left panel)

Chemiluminescence detection

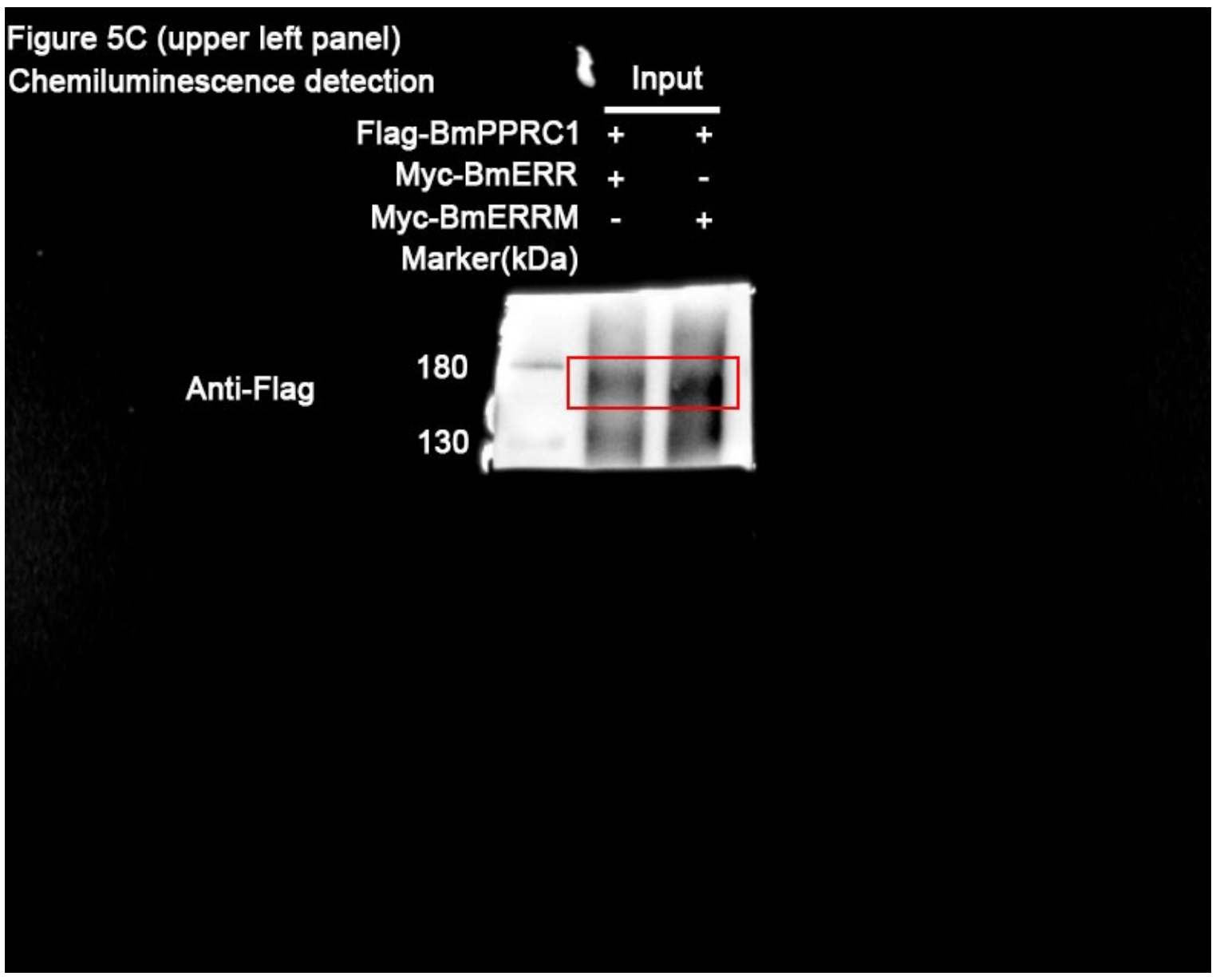

Figure 5C (upper right panel)

Chemiluminescence detection

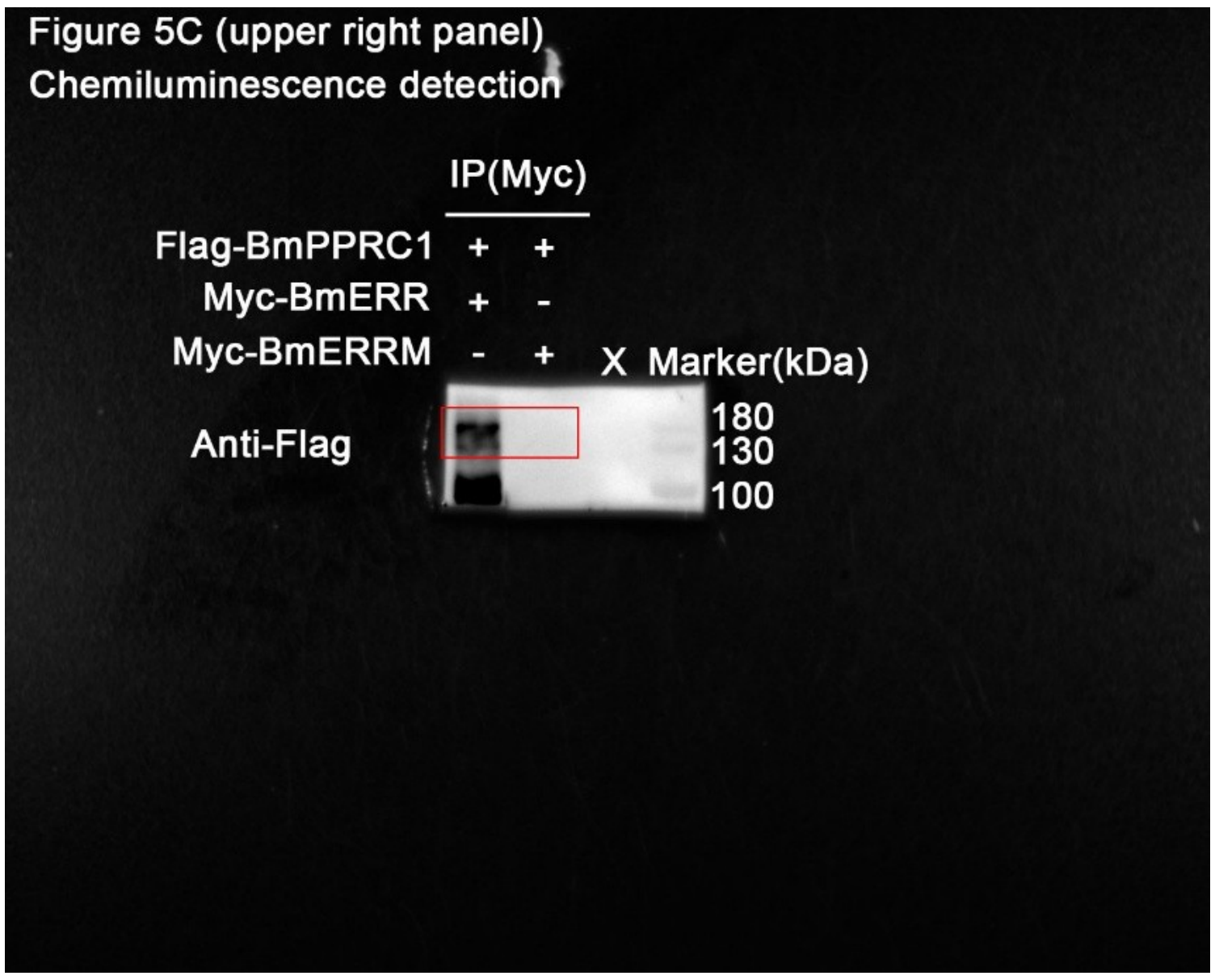

Figure 5C (lower left panel)

Chemiluminescence detection

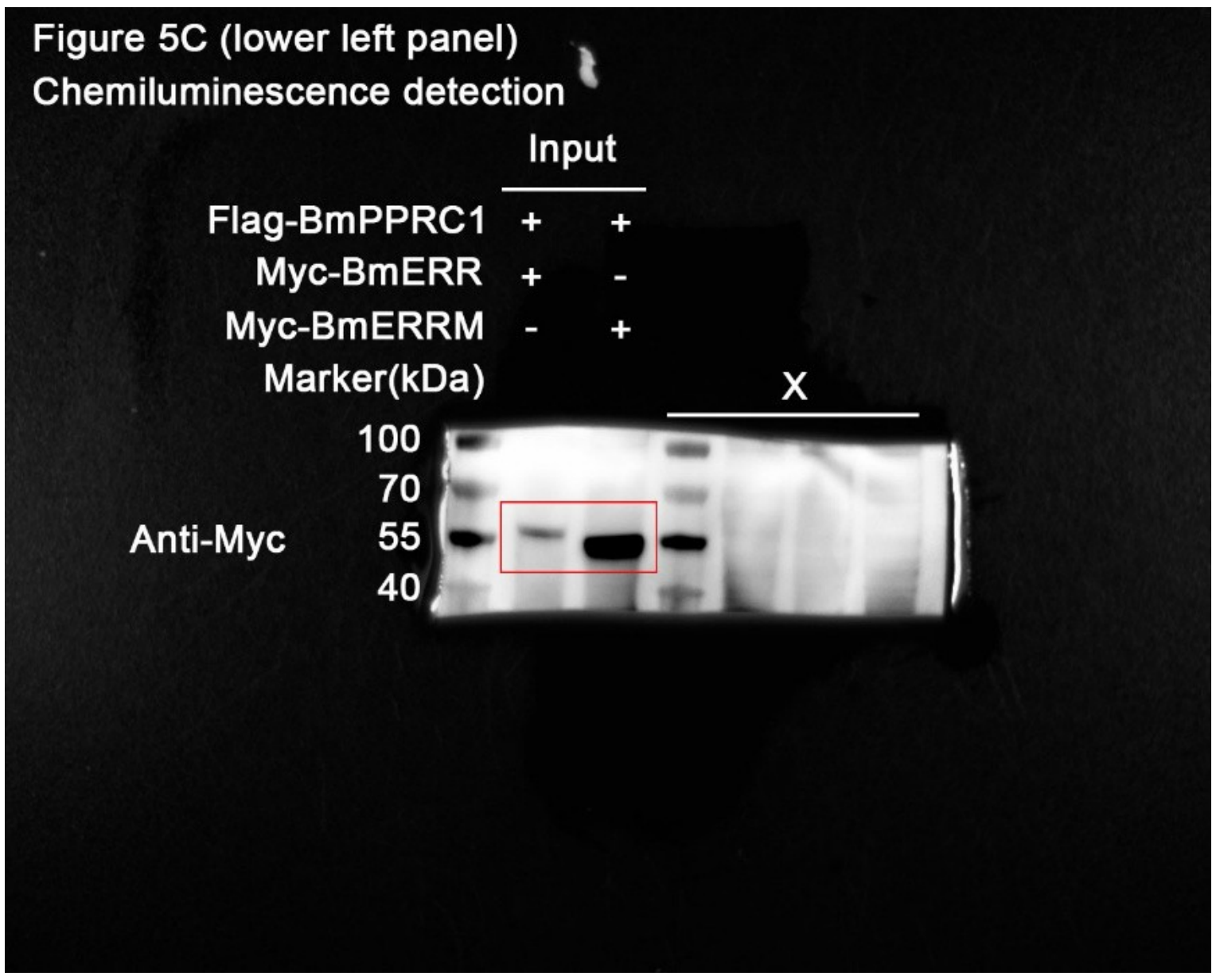

Figure 5C (upper right panel)

Chemiluminescence detection

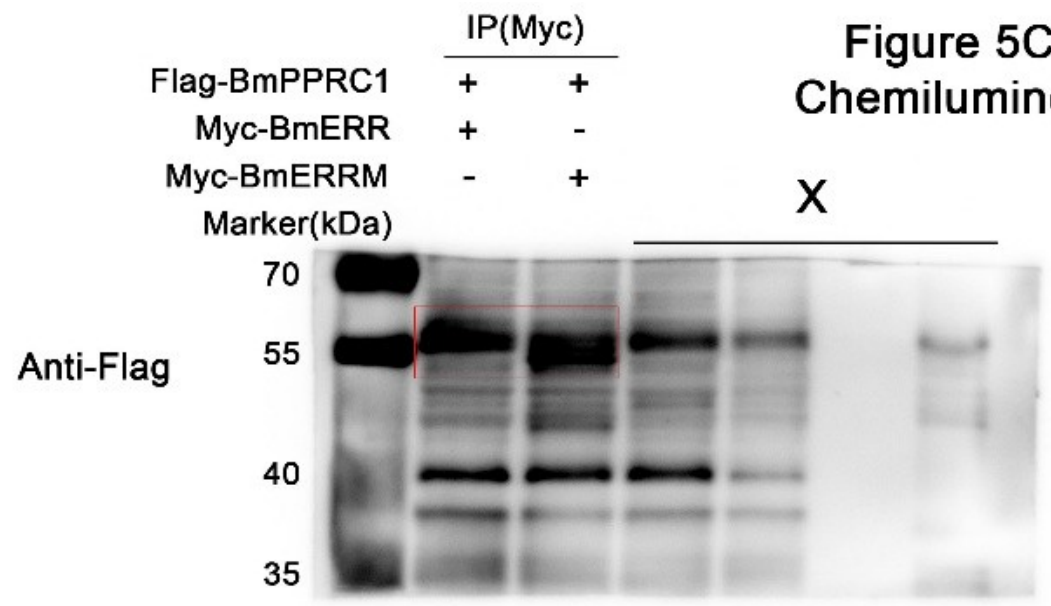

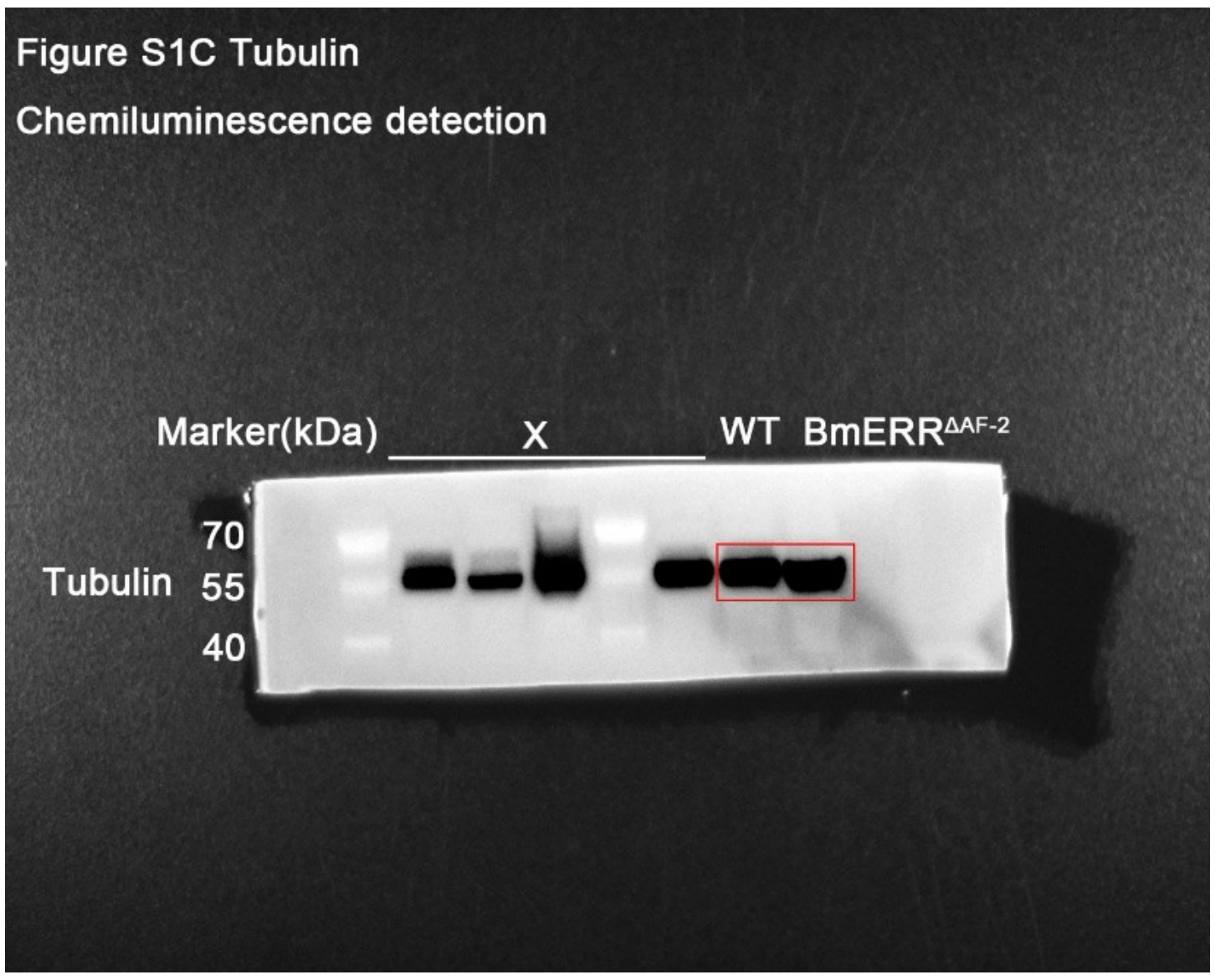
Figure S1C Tubulin
Chemiluminescence detection
Marker(kDa)
X
WT
BmERR$^{\Delta AF-2}$
70
Tubulin
55
40

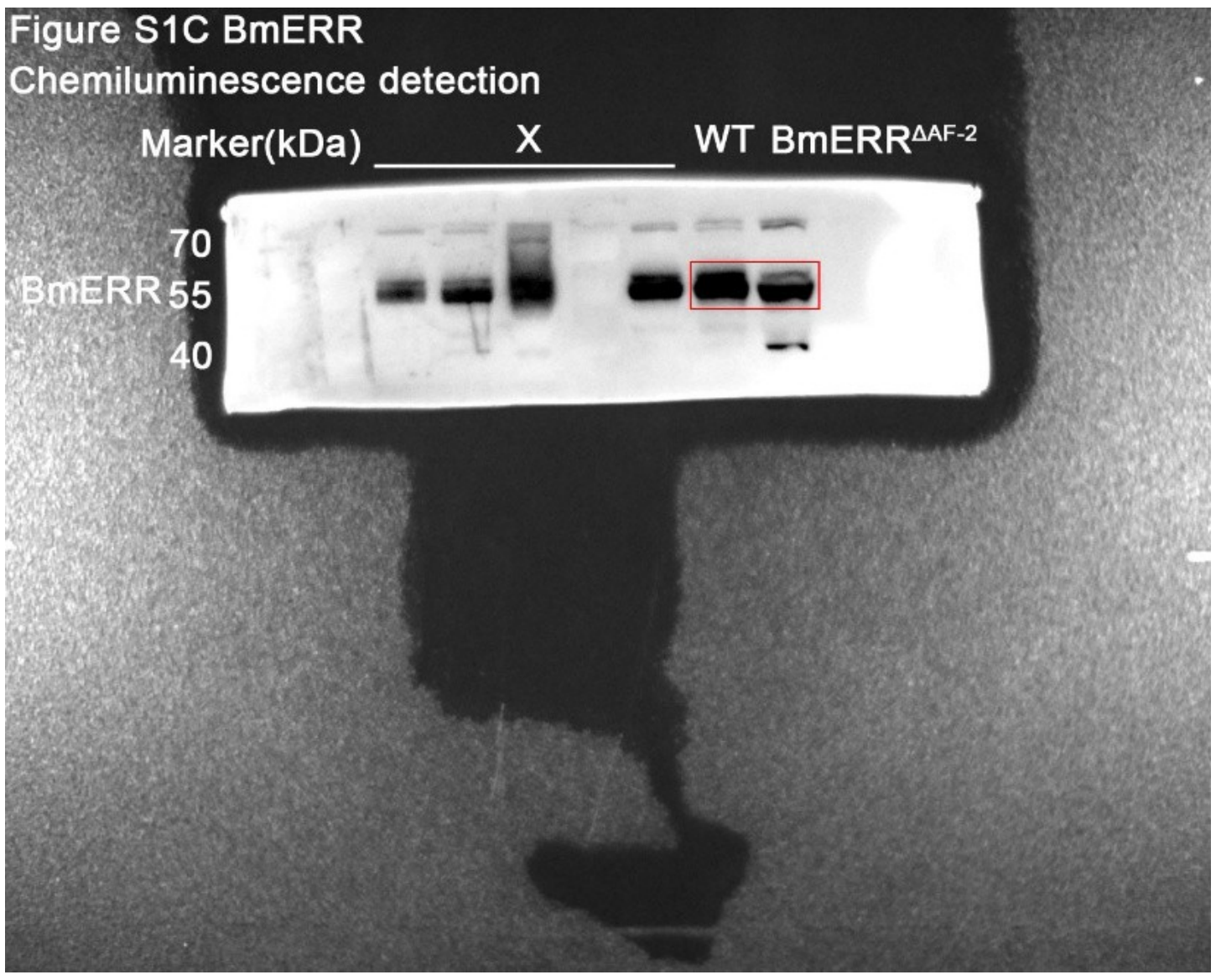
Figure S1C BmERR
Chemiluminescence detection
Marker(kDa)
X
WT
BmERR$^{\Delta AF-2}$
70
BmERR
55
40

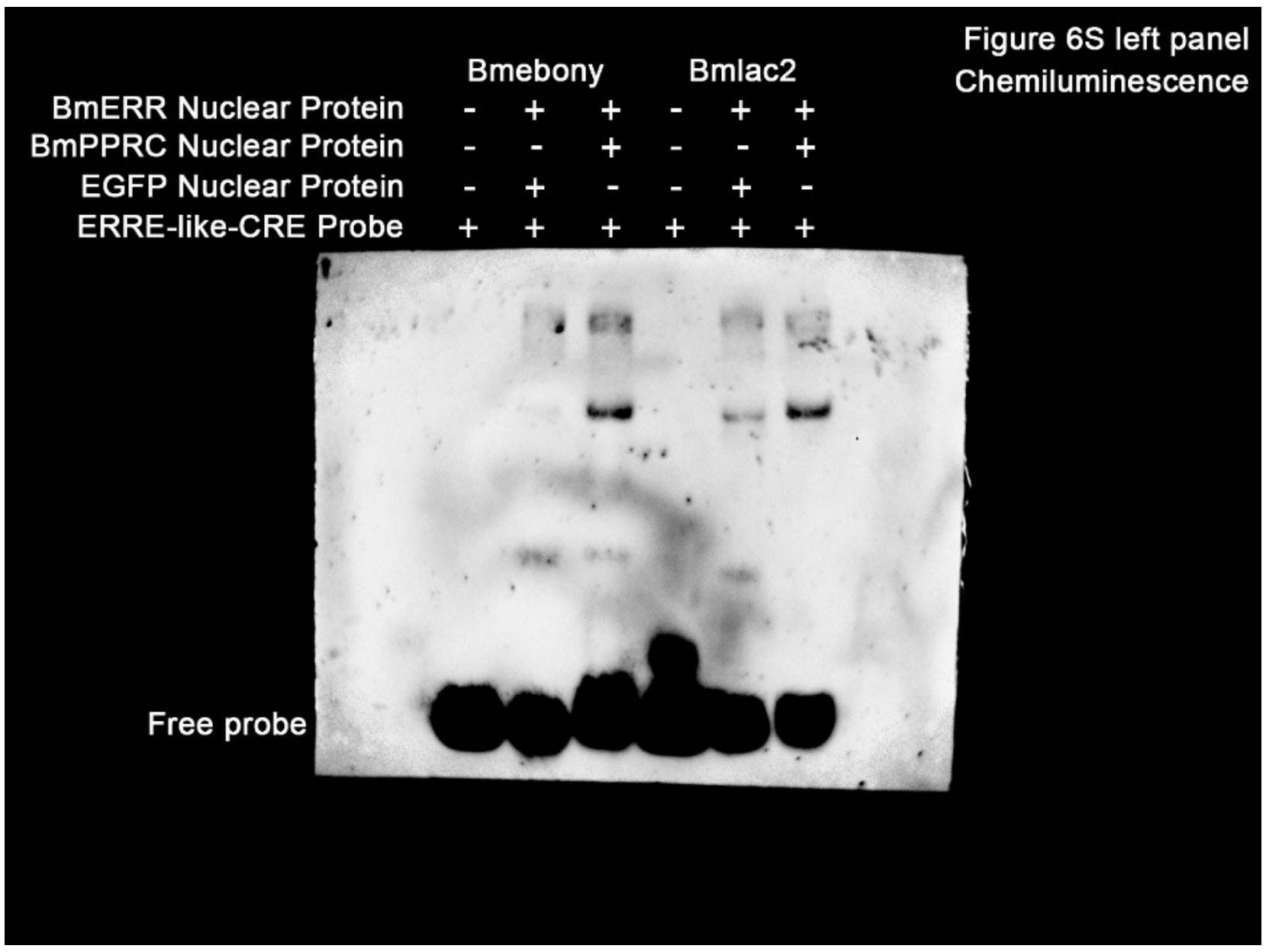
Figure 6S left panel
Chemiluminescence
Bmebony
Bmlac2
BmERR Nuclear Protein - + + - + +
BmPPRC Nuclear Protein - - + - - +
EGFP Nuclear Protein - + - - + -
ERRE-like-CRE Probe + + + + + +
Free probe

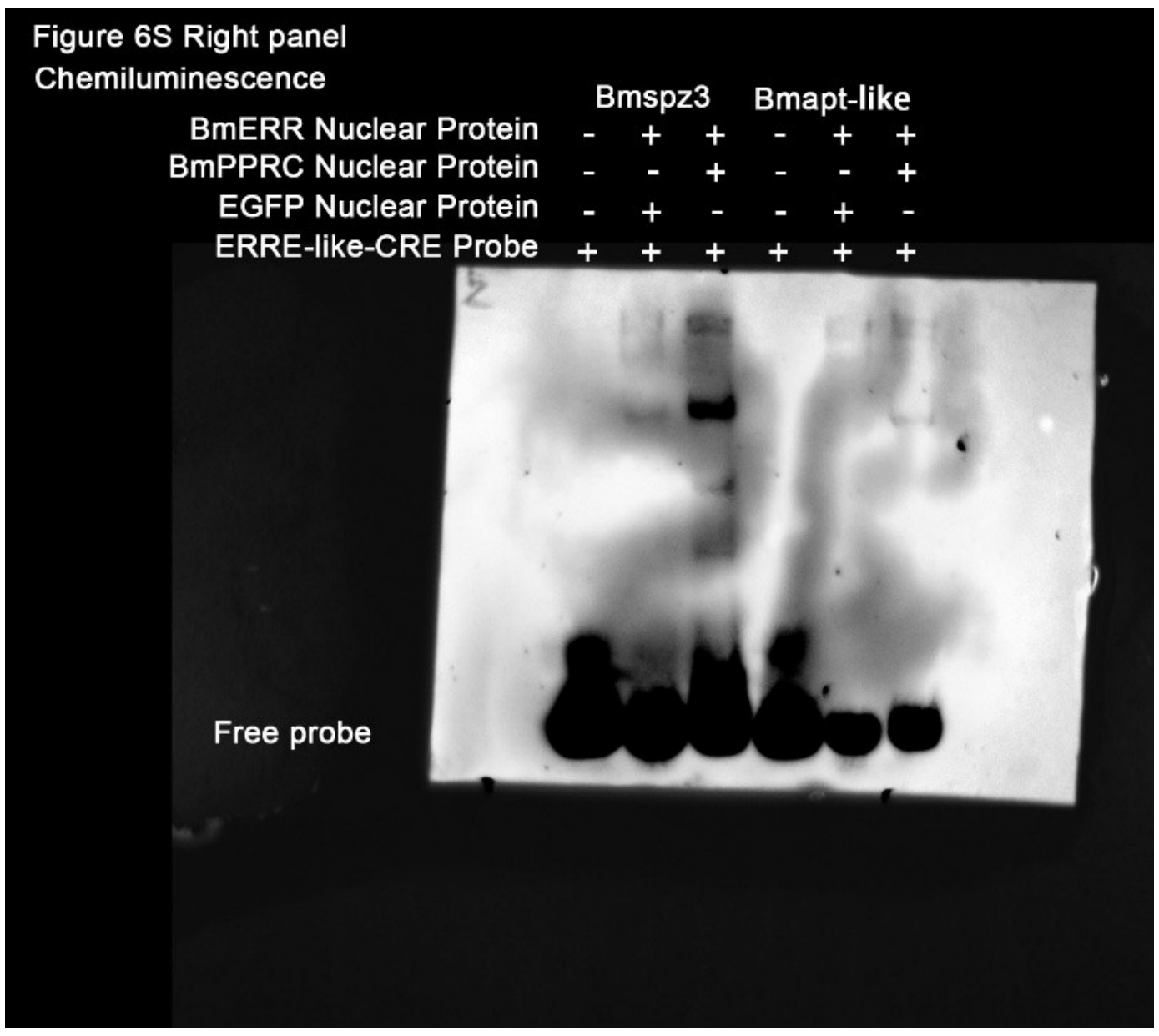
Figure 6S Right panel
Chemiluminescence
Bmspz3
Bmapt-like
BmERR Nuclear Protein - + + - + +
BmPPRC Nuclear Protein - - + - - +
EGFP Nuclear Protein - + - - + -
ERRE-like-CRE Probe + + + + + +
Free probe

Raw images--Photograph

Figure 2A

Digital camera

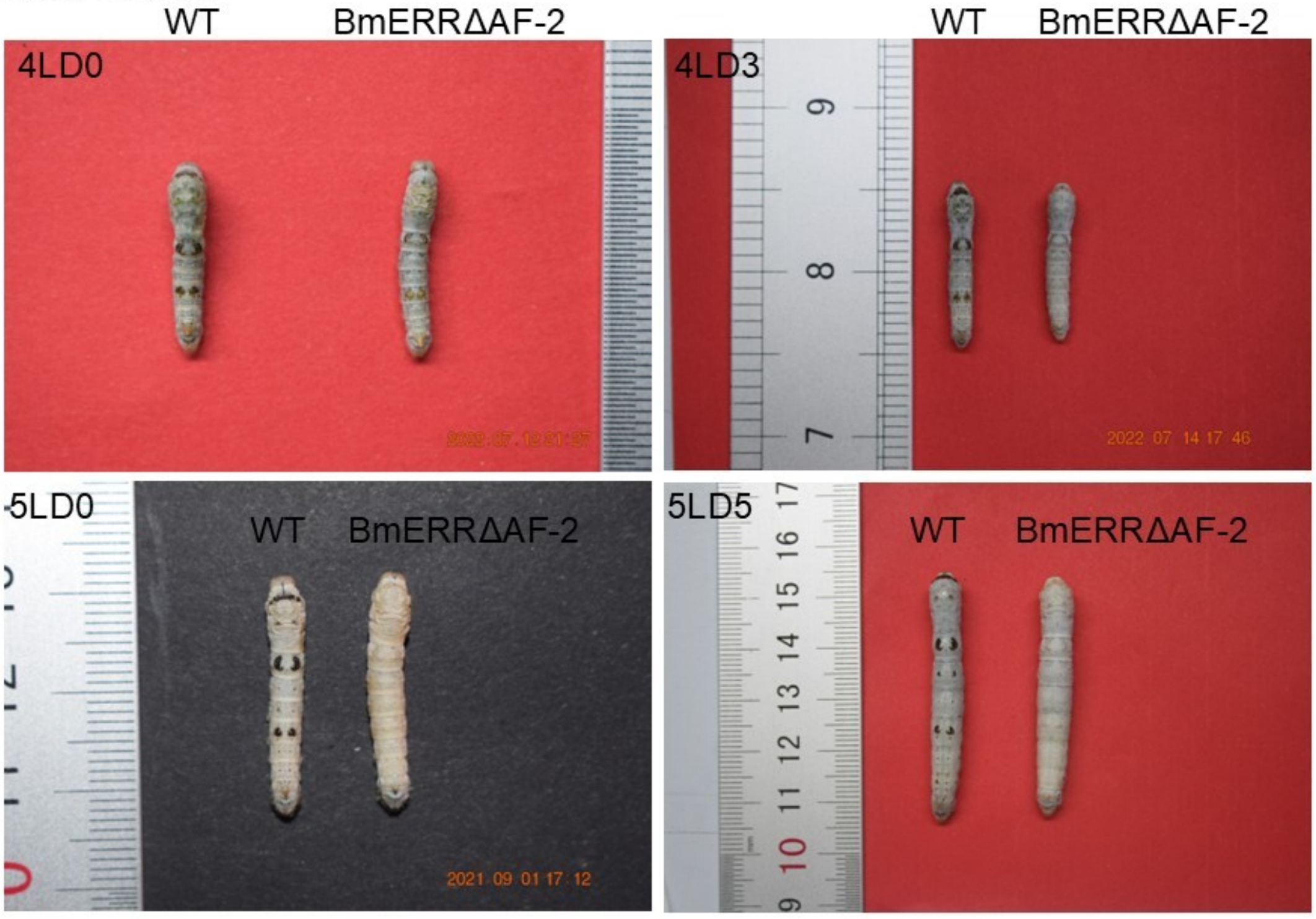

Figure 3B

Digital camera

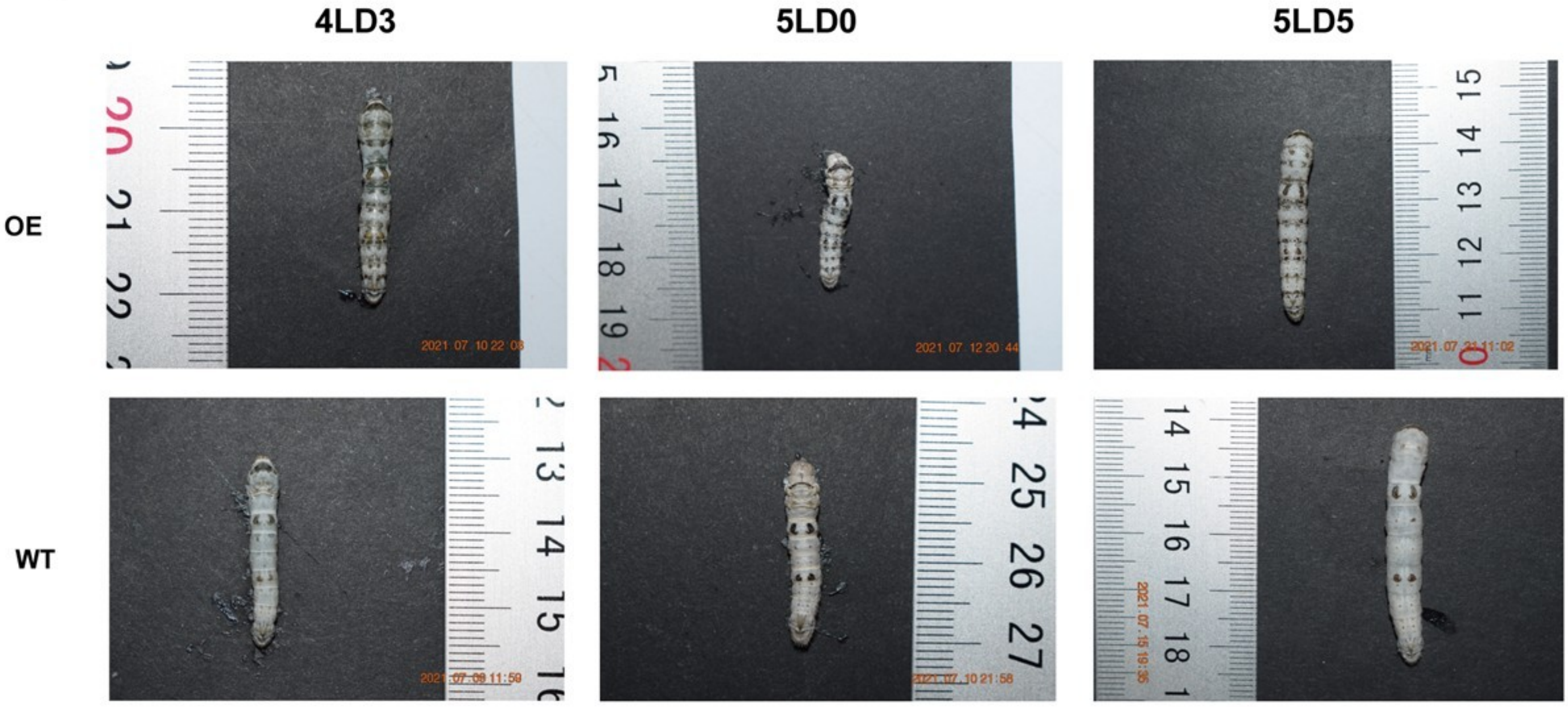

Figure S2B

Digital camera

WT BmERRΔAF-2

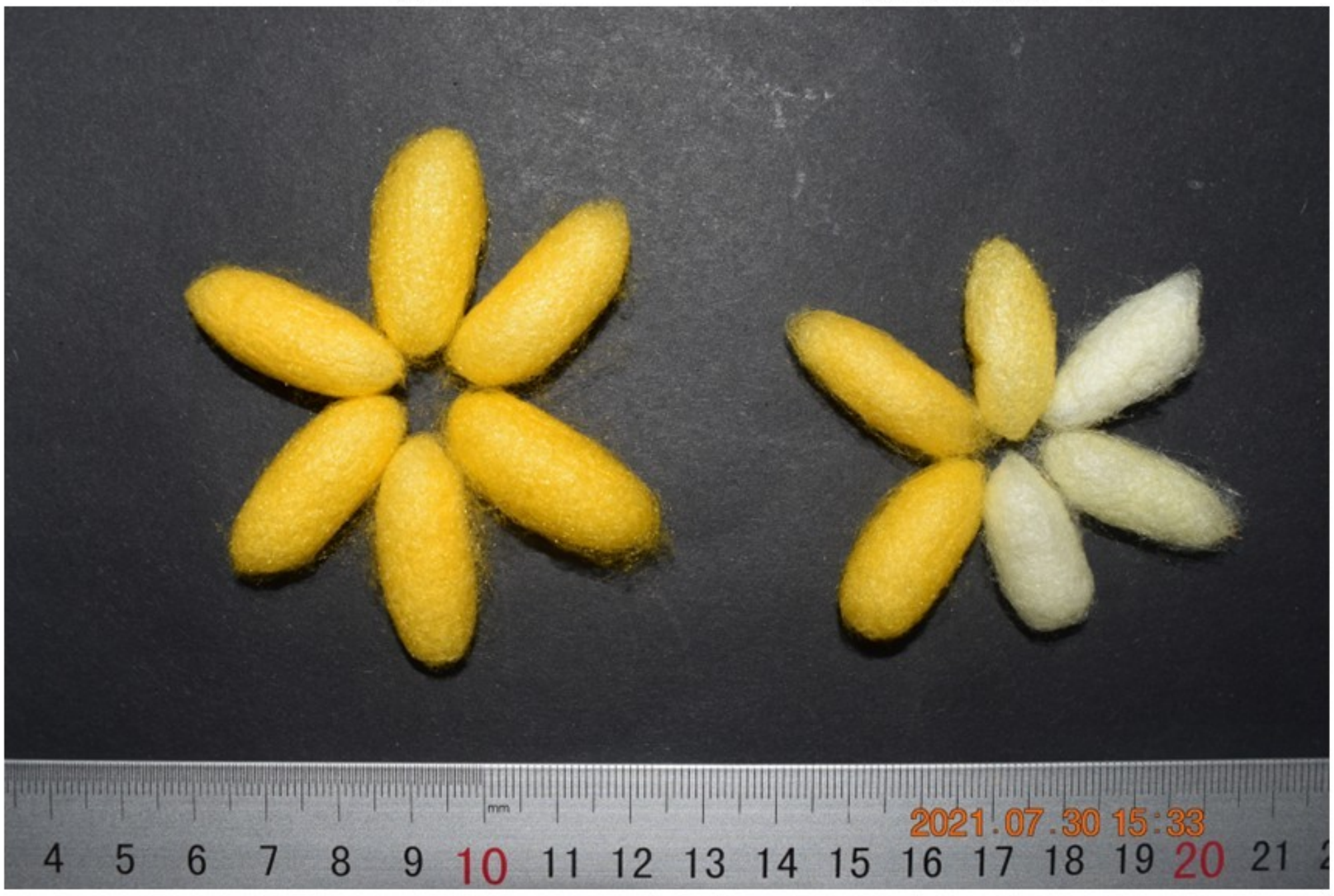

Supplement: Supplementary file 1 [file pgen.1012339.s008.pdf]
