# Supplementary material for: The AF-2 domain of estrogen-related receptor interacts with PPRC1 to modulate larval epidermal marking in Bombyx mori
Source: PLoS Genet. 2026 Oct 12;22(10):e1012339. doi: 10.1371/journal.pgen.1012339 (PMC13657282; doi:10.1371/journal.pgen.1012339)
Supplement: Supplementary file 2 [file pgen.1012339.s007.docx]

| **Primer name** | **Purpose** | **5′-3′** | **Sequence** | **Gene ID** | **Remark** |
| --- | --- | --- | --- | --- | --- |
| AF-2 gRNA | Construction of transgenic vectors | F | AAGTGCGTGGCCGTGCGCCGCTTCT |  |  |
|  |  | R | AAACAGAAGCGGCGCACGGCCACGC |  |  |
| pGL3-BmebonyP | Vector for cell expression | F | cgagctcCTAGCAAGAGAAGTGCTTCGC |  | Sac I |
|  |  | R | cccaagcttCGCAAATGCACCGTAAACACAG |  | Hind Ⅲ |
| pGL3-Bmlac2P |  | F | ccgctcgagCCTAACAATGTCACAATGACTGCGC |  | Xho I |
|  |  | R | cccaagcttGCCAGTCGCTCTGCATTTGCTGA |  | Hind Ⅲ |
| pGL3-Bmapt-likeP |  | F | ctagctagcGCGTAATTACTGTTGGTAGGACC |  | Kpn I |
|  |  | R | ccgctcgagCACTCAAGTACTTGTGCCAAATG |  | Xho I |
| pGL3-Bmspz3P |  | F | ggggtaccGTGCTGATCCTGATGTCGTTCACC |  | Nhe I |
|  |  | R | ccgctcgagATGGCGGCAAGACATGACATGCGC |  | Xho I |
| 1180-myc-BmERR |  | F | aggattggtggatccATGGAGCAGAAACTCATCTCTGAAGAGGATCTGATGTCCGCAGTCAGTGG |  |  |
|  |  | R | agttgtagcggccgcTTACCGCAGACAGGCCTCGA |  |  |
| 1180-myc-BmERRM |  | F | aggattggtggatccATGGAGCAGAAACTCATCTCTGAAGAGGATCTGATGTCCGCAGTCAGTGG |  |  |
|  |  | R | agttgtagcggccgcTTAGTCCGCGTGGCGCAGCGCCGGCAGC |  |  |
| 1180-Flag-BmPPRC1 |  | F | aggattggtggatccATGGATTACAAGGATGACGACGATAAGGAGTCCCACATACTGAACAC |  |  |
|  |  | R | agttgtagcggccgcTCAAGGCTTGGTGGCGTCGCCGC |  |  |
| *BmPPRC1* | qRT-PCR | F | GCAACAGTCGAATACTCAACTC | 101736112 | 132 bp |
|  |  | R | CGTCGTCACTTTGATTAGTCAC |  |  |
| *BmERR* |  | F | CGCCGACCTGTACGACC | 101742160 | 259 bp |
|  |  | R | CACGCCCGACACCTGTAGAAA |  |  |
| *BmTIF4A* |  | F | TTCGTACTGGCTCTTCTCGT | 692781 | 196 bp |
|  |  | R | CAAAGTTGATAGCAATTCCCT |  |  |
| *BmTH* |  | F | TTCGCCATCAAGAAATCCTAC | 100270767 | 255 bp |
|  |  | R | GAGGTGACCATTTTTCGTTTCA |  |  |
| *BmDDC* |  | F | TTATGAAAGGCGTTGAGAAAGC | 692675 | 135 bp |
|  |  | R | AAAGAGGATCAACGTTGAAAGC |  |  |
| *Bmebony* |  | F | CTTTGAACTTCGACGTGAGAGA | 100270765 | 83 bp |
|  |  | R | CACGTACTCCAAAAATCCCATG |  |  |
| *Bmtan* |  | F | GTACGGCAGTATGTGAGATACA | 100384889 | 178 bp |
|  |  | R | GTATCAGTTTTGTATTCGCGCT |  |  |
| *BmAA-NAT* |  | F | AAGTAGACTACACGGACAAGTC | 780849 | 136 bp |
|  |  | R | ATCTCGAGAACAGAGTCACAAG |  |  |
| *Bmspz3* |  | F | CGAGCTATTGTGAACACTATGC | 101743559 | 117 bp |
|  |  | R | GTGCCATTTGTATTTTTGCTCG |  |  |
| *Bmapt-like* |  | F | ACGATCACATAAAGCACGAATG | 101739914 | 143 bp |
|  |  | R | GAATATCTCCTCCTTCTGCCTC |  |  |
| ERRE -CRE  (*Bmapt-like*) | EMSA | F | GATATCTATGACCTCCAGTAACC | 3′–ends biotin labeled | |
|  |  | R | GGTTACTGGAGGTCATAGATATC |  |  |
| ERRE -CRE  (*Bmebony*) |  | F | GAATAGAATAACCTTGATAGAATTG |  |  |
|  |  | R | CAATTCTATCAAGGTTATTCTATTC |  |  |
| ERRE -CRE  (*Bmlac2*) |  | F | GCGCCATCCACCTTGAGATATAA |  |  |
|  |  | R | TTATATCTCAAGGTGGATGGCGC |  |  |
| ERRE -CRE  (*Bmspz3*) |  | F | AACCAGCCAGACCTTGTCCAACA |  |  |
|  |  | R | TGTTGGACAAGGTCTGGCTGGTT |  |  |
